# Neighborhood social vulnerability and disparities in time to kidney cancer surgical treatment and survival in Arizona

**DOI:** 10.1002/cam4.7007

**Published:** 2024-02-24

**Authors:** Celina I. Valencia, Patrick Wightman, Kristin E. Morrill, Chiu‐Hsieh Hsu, Hina Arif‐Tiwari, Eric Kauffman, Francine C. Gachupin, Juan Chipollini, Benjamin R. Lee, David O. Garcia, Ken Batai

**Affiliations:** ^1^ Department of Family and Community Medicine, College of Medicine – Tucson The University of Arizona Tucson Arizona USA; ^2^ Center for Population Health Sciences The University of Arizona Tucson Arizona USA; ^3^ Community and Systems Health Science Division, College of Nursing The University of Arizona Tucson Arizona USA; ^4^ Department of Epidemiology and Biostatistics The University of Arizona Tucson Arizona USA; ^5^ Department of Medical Imaging The University of Arizona Tucson Arizona USA; ^6^ Department of Urology Roswell Park Comprehensive Cancer Center Buffalo New York USA; ^7^ Department of Urology The University of Arizona Tucson Arizona USA; ^8^ Department of Health Promotion Sciences The University of Arizona Tucson Arizona USA; ^9^ Department of Cancer Prevention and Control Roswell Park Comprehensive Cancer Center Buffalo New York USA

**Keywords:** cancer health disparities, neighborhood factors, renal cancer, social determinants of health, treatment disparities

## Abstract

**Background:**

Hispanics and American Indians (AI) have high kidney cancer incidence and mortality rates in Arizona. This study assessed: (1) whether racial and ethnic minority patients and patients from neighborhoods with high social vulnerability index (SVI) experience a longer time to surgery after clinical diagnosis, and (2) whether time to surgery, race and ethnicity, and SVI are associated with upstaging to pT3/pT4, disease‐free survival (DFS), and overall survival (OS).

**Methods:**

Arizona Cancer Registry (2009–2018) kidney and renal pelvis cases (*n* = 4592) were analyzed using logistic regression models to assess longer time to surgery and upstaging. Cox‐regression hazard models were used to test DFS and OS.

**Results:**

Hispanic and AI patients with T1 tumors had a longer time to surgery than non‐Hispanic White patients (median time of 56, 55, and 45 days, respectively). Living in neighborhoods with high (≥75) overall SVI increased odds of a longer time to surgery for cT1a (OR 1.54, 95% CI: 1.02–2.31) and cT2 (OR 2.32, 95% CI: 1.13–4.73). Race and ethnicity were not associated with time to surgery. Among cT1a patients, a longer time to surgery increased odds of upstaging to pT3/pT4 (OR 1.95, 95% CI: 0.99–3.84). A longer time to surgery was associated with PFS (HR 1.52, 95% CI: 1.17–1.99) and OS (HR 1.63, 95% CI: 1.26–2.11). Among patients with cT2 tumor, living in high SVI neighborhoods was associated with worse OS (HR 1.66, 95% CI: 1.07–2.57).

**Conclusions:**

High social vulnerability was associated with increased time to surgery and poor survival after surgery.

## Graphical abstract

This study investigated whether racial and ethnic minority patients and patients from neighborhoods with high social vulnerability index experience a longer time to surgery for treatment of kidney cancer after clinical diagnosis, and whether a time to surgery, race and ethnicity, and SVI are associated with upstaging to pT3/pT4, disease‐free survival, and overall survival in Arizona. This study found that high social vulnerability is associated with increased time to surgery and poor survival after surgery.

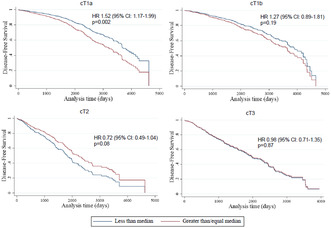

## 1 INTRODUCTION

Kidney cancer (KCa) is one of the top 10 most common cancers in the United States, and its incidence is increasing nationally and globally.^1^, ^2^, ^3^ Surgery (nephrectomy) is most often the primary treatment for localized KCa without neoadjuvant treatment. Decisions for surgical treatment are made based on tumor size (clinical stage), complexity (solitary vs. multiple tumors), renal function, overall health conditions, and age.^4^, ^5^, ^6^, ^7^ Active surveillance is recommended for elderly patients with comorbidities and patients with a small tumor (<4 cm, clinical stage 1a [cT1a]). Partial nephrectomy is recommended for clinical stage 1 (T1, <7 cm) tumors. Partial nephrectomy is also recommended for patients with limited renal function, solitary, or bilateral tumors. Radical nephrectomy is generally performed for patients with clinical stage 2 (cT2) and 3 (cT3) tumors. While active surveillance for cT1a tumor is shown to be safe, for larger tumors, there is no clear guideline for how quickly surgical treatment needs to be performed after clinical diagnosis.

Disparities in receipt and type of surgical treatment have been previously reported, but disparities in time to surgical treatment have not been explored.^8^, ^9^, ^10^, ^11^ Surgery wait time of up to 3 months for early‐stage KCa may not impact survival after surgical treatment, but prolonged time to surgery is associated with reduced survival.^12^, ^13^, ^14^ Disparities in time to treatment initiation exist for many cancer types in racial and ethnic minority populations, including KCa. Racial and ethnic minority groups with high mortality rates often experience a longer gap between diagnosis to treatment initiation.^15^, ^16^, ^17^, ^18^, ^19^ A better understanding of disparities in time to treatment initiation is necessary to develop programs or recommendations to reduce current patterns of KCa disparities.

Neighborhood‐level attributes have been associated with numerous cancer health disparities. For breast,^20^, ^21^ prostate,^22^ lung,^23^ cervical,^24^ and liver^25^, ^26^ cancers, living in low socioeconomic status (SES) neighborhoods is linked to disease disparities. A suggested pathway shaping these disparities is the relationship between neighborhood‐level disadvantage and cancer treatment delay, which has been previously considered for breast,^18^ colorectal,^16^ and liver^19^ cancers. The impacts of neighborhood‐level disadvantage on cancer treatment delay are particularly pronounced among individuals from racial and ethnic minority populations.^16^, ^27^ Social vulnerability index (SVI) is a publicly available measurement of neighborhood characteristics. The SVI was developed by the Centers for Disease Control and Prevention (CDC) using U.S. Census data and American Community Survey data to identify socially vulnerable communities for disaster responses.^28^ The SVI is also useful to assess impacts of neighborhood factors influencing diagnosis, treatment, survivorship care, and outcomes of many cancer types.^29^ Compared to low SVI neighborhoods, high SVI neighborhoods are shown to have lower cancer screening rates and higher cancer mortality rates.^30^, ^31^, ^32^ Living in high SVI neighborhoods is also associated with foregoing curative surgical treatment for hepatocellular carcinoma.^33^ However, despite the usefulness of SVI to assess impacts of neighborhood characteristics on cancer care and outcomes, SVI has not been previously used in KCa studies.

To better understand these racial and ethnic differences in KCa survival, the current study examined the relationships between neighborhood‐level factors, time to treatment initiation, and cancer upstaging and survival in Arizona. This study assessed: (1) whether racial and ethnic minority patients and patients from neighborhoods with high social vulnerability as measured by the SVI experience a longer time to surgical treatment after clinical diagnosis, and (2) whether time to surgery, race and ethnicity, and neighborhood social vulnerability are associated with adverse pathology (upstaging to pT3/pT4), disease‐free survival (DFS), and overall survival (OS). The state of Arizona is uniquely situated to examine neighborhood factors related to a longer time from diagnosis to surgical treatment given its high numbers of rural American Indian (AI) reservations and geographic regions on the US/Mexico border. This study extends upon previously reported evidence that AI and Mexican American patients have an elevated risk of KCa mortality compared to non‐Hispanic White (NHW) patients.^34^

## 2 MATERIALS AND METHODS

### 2.1 KCa case data

Arizona Cancer Registry (ACR) data for kidney and renal pelvis cases (International Classification of Diseases for Oncology‐10‐CM C649 and C659) diagnosed and/or treated between January 1, 2009, and December 31, 2018 were obtained. Adult patients (aged ≥20 years) who (1) underwent surgical treatment, including local ablation, partial nephrectomy, radical/total nephrectomy, and unknown surgical treatment and (2) had data available on clinical tumor stage 1, 2, and 3 (cT1, cT2, and cT3) were included in the study (*n* = 4592) (Figure S1). Cases without clinical stages and cases with clinical tumor stage 4 or metastatic cancer were excluded. Approval to conduct this study was obtained from the Arizona Department of Health Services Human Subject Review Board.

### 2.2 Outcome variables

This study assessed three outcomes: (1) time (in days) from date of clinical diagnosis to surgical treatment; (2) adverse pathology which included any upstaging and upstaging to pathological stage 3 (pT3) or 4 (pT4) from cT1a, cT1b, and cT2; and (3) survival measured as DFS and OS. Among patients with cT1, cT2, and cT3 tumors, patients for which there was no clinical diagnosis date available prior to surgery (i.e., where time to surgery = 0) were excluded (*n* = 2019) initially. We then included these cases to assess how removing them may have affected analysis results. Because there is no clear clinical guideline for appropriate time between clinical diagnosis and time to surgical treatment, the median time to surgery from diagnosis was calculated separately for each clinical tumor stage to define a longer time to surgery (>median time to surgery). We also assessed whether race and ethnicity and SVI were associated with >1 month and >3 months from the date of clinical diagnosis to surgical treatment. DFS and OS was evaluated using time in days from date of surgery to date of death, recurrence (for DFS only), or last follow‐up.

### 2.3 Exposure variables

Race and ethnicity reported to ACR was used and categorized into five groups (NHW, non‐Hispanic Black [NHB], AI, Hispanics, and Other for individuals with unknown or mixed race and ethnicity). The SVI was used as a measurement of neighborhood characteristics and linked to census tract geocode of each patient. The overall SVI, a composite measure of 15 factors, groups these factors into four themes: (1) SES, (2) household composition and disability, (3) minority status and language, and (4) housing and transportation. The SVI ranges from 0 (least vulnerable) to 1 (most vulnerable) and is reported using percentile. For overall SVI, the scores were grouped into four categories (<25th, 25th–49th, 50–74th, and ≥75th percentile). Each individual SVI theme was grouped into two categories (<75th and ≥75th percentile). For analysis stratified by SVI, overall SVI, and each individual SVI theme were grouped to <50th and ≥50th percentile.

### 2.4 Covariates

Adjusted models included age, sex, insurance type, region/county, and urban/nonurban residence. Insurance type was categorized into Private, Medicaid, Medicare, and Other which included uninsured, unknown, military/Veterans Affairs, and Indian Health Services. Private insurance was used as the reference category. Arizona counties were divided into three groups based on geographic location: Central/East (Apache, Cochise, Coconino, Gila, Graham, Greenlee, Navajo, Pinal, and Yavapai), West (La Paz, Mohave, and Yuma), and South/Central (Pima and Santa Cruz). The largest county, Maricopa, served as the reference for geographic analyses. The U.S. Department of Agriculture 2010 Rural–Urban Commuting Area codes based on the census tract were used to categorize residential areas into urban (metropolitan, urban cities, and suburbs) and nonurban (large rural city/town and small isolated rural town).

### 2.5 Statistical analysis

Percentages and medians, including interquartile ranges (IQRs), were used to assess sociodemographic and clinical characteristics. Differences in clinical characteristics and SVI were tested using chi‐squared tests for categorical variables and Kruskal–Wallis tests for continuous variables. Logistic regression models were used to assess the associative relationship of SVI and race and ethnicity with a longer time to surgical treatment and adverse pathology. Cox regression hazard models were used to assess whether a longer time to surgery and SVI were associated with DFS and OS. Adjusted models were run first using overall SVI, and then using each individual SVI theme. Statistical analysis was performed separately for clinical tumor stage 1 (cT1a and cT1b), 2 (cT2), and 3 (cT3) patients. Clinical tumor stage 1 was separated into cT1a and cT1b, because patients with cT1a tumor may be eligible for active surveillance.^35^ Heterogeneity in associations between race and ethnicity and time to surgery was assessed by stratifying cases by SVI. Ad hoc analyses were performed to assess potential bias in registry data regarding patients for which a diagnosis date was not available prior to surgery.

## 3 RESULTS

### 3.1 Characteristics of patients

**FIGURE 1:**
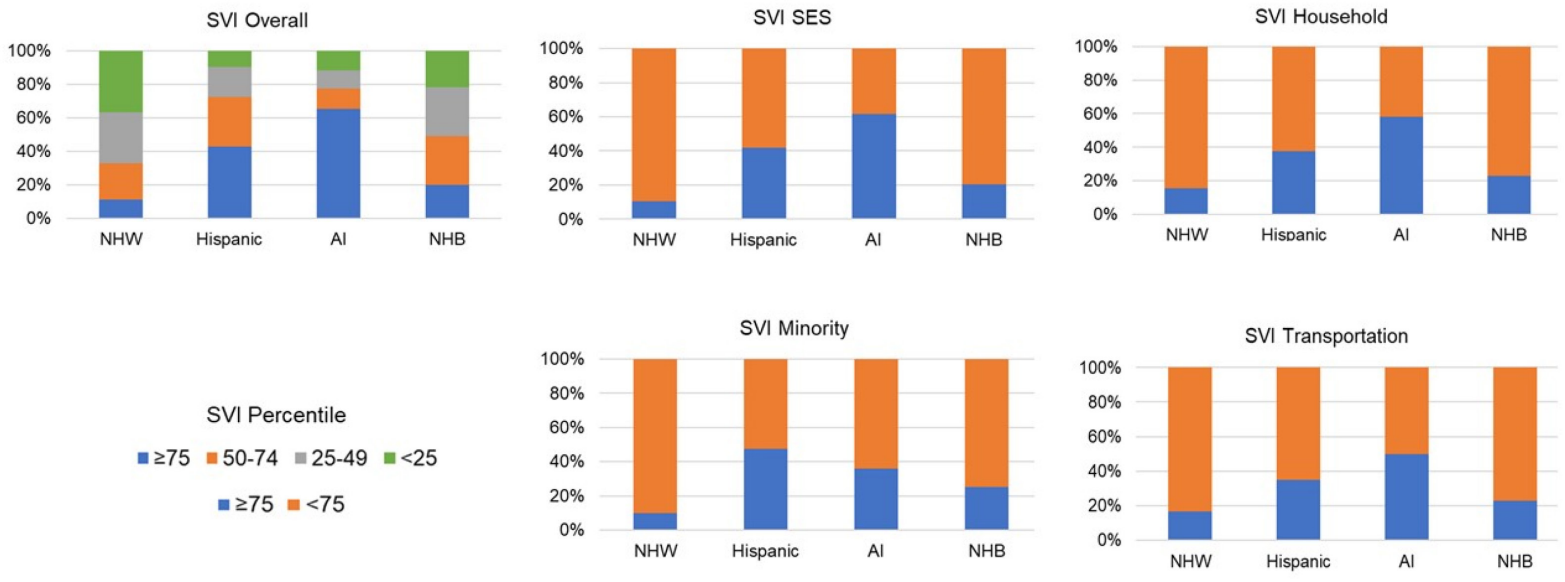
Social vulnerability index (SVI) of kidney cancer patients' residence by race and ethnicity.

There was a total of 4592 cases with cT1, cT2, or cT3 tumors (Table 1). The largest racial and ethnic group was NHW (73.3%) followed by Hispanic (16.6%). Combined cases of AI and NHB patients represented a total of 8.5% of the study sample (4.8% and 3.7%, respectively). Age, sex, insurance type, region/county, and urban/nonurban residence of patients differed significantly by race and ethnicity. A greater proportion of Hispanic and AI patients belonged to younger age groups and had Medicaid coverage compared to NHW and NHB patients. There were also racial and ethnic differences in geographic distribution. The proportion of Hispanic patients living in the South/Central counties was higher than that of NHW patients (15.1% vs. 7.7%), while the proportion of AI patients living in Central/East counties was higher than that of NHW patients (42.7% vs. 12.3%). AI patients were more likely to live in nonurban areas than NHW patients (27.3% vs. 10.4%). Hispanic and AI patients were more likely than NHWs and NHBs to come from neighborhoods with high SVI scores (Figure 1; Table S1).

**TABLE 1:** Characteristics of patients by race and ethnicity.

	Total	NHW	Hispanic	AI	NHB	Other	*p*
*n* (%)	4592 (100)	3364 (73.3)	764 (16.6)	220 (4.8)	171 (3.7)	73 (1.6)	
Age, *n* (%)							
Age <50	716 (15.6)	426 (12.7)	174 (22.8)	64 (29.1)	35 (20.5)	17 (23.3)	<0.001
Age 50–59	1443 (31.4)	679 (20.2)	228 (29.8)	60 (27.3)	48 (28.1)	29 (39.7)	
Age 60–69	1044 (22.7)	1049 (31.2)	210 (27.5)	63 (28.6)	55 (32.2)	12 (16.4)	
Age ≥70	1389 (30.2)	1210 (36.0)	152 (19.9)	33 (15.0)	33 (19.3)	15 (20.5)	
Sex, *n* (%)							
Male	2968 (64.6)	2214 (65.8)	464 (60.7)	132 (60.0)	112 (65.5)	46 (63.0)	0.053
Female	1624 (35.4)	1150 (34.2)	300 (39.3)	88 (40.0)	59 (34.5)	27 (37.0)	
Insurance type, *n* (%)							
Private	1681 (36.6)	1253 (37.2)	268 (35.1)	69 (31.4)	56 (32.7)	35 (47.9)	<0.001
Medicaid	425 (9.3)	192 (5.7)	165 (21.6)	36 (16.4)	23 (13.5)	9 (12.3)	
Medicare	1914 (41.7)	1550 (46.1)	206 (27.0)	65 (29.5)	73 (42.7)	20 (27.4)	
Other	572 (12.5)	369 (11.0)	125 (16.4)	50 (22.7)	19 (11.1)	9 (12.3)	
Region/county, *n* (%)							
Maricopa	3016 (65.7)	2277 (67.7)	452 (59.2)	98 (44.5)	133 (77.8)	56 (76.7)	<0.001
West	547 (11.9)	415 (12.3)	110 (14.4)	14 (6.4)	4 (2.3)	4 (5.5)	
Central East	619 (13.5)	414 (12.3)	87 (11.4)	94 (42.7)	21 (12.3)	3 (4.1)	
South Central	410 (8.9)	258 (7.7)	115 (15.1)	14 (6.4)	13 (7.6)	10 (13.7)	
Urban/nonurban residence, *n* (%)							
Urban	4100 (89.3)	3015 (89.6)	691 (90.4)	160 (72.7)	164 (95.9)	70 (95.9)	<0.001
Nonurban	492 (10.7)	349 (10.4)	73 (9.6)	60 (27.3)	7 (4.1)	3 (4.1)	
Clinical stage, *n* (%)							
cT1	3405 (74.2)	2491 (74.0)	557 (72.9)	162 (73.6)	138 (80.7)	57 (78.1)	0.47
cT2	600 (13.1)	447 (13.3)	100 (13.1)	25 (11.4)	19 (11.1)	9 (12.3)	
cT3	587 (12.8)	426 (12.7)	107 (14.0)	33 (15.0)	14 (8.2)	7 (9.6)	
Time to surgery in days, median (IQR)							
cT1 (All)	48 (28, 75)	45 (27, 72)	56 (34, 89)	55 (32, 75)	49 (27, 75)	43 (32, 74)	0.007
cT1a	51 (31, 78)	49 (29, 76)	58 (37, 92)	56 (38, 71)	52 (30, 74)	43 (35, 74)	0.19
cT1b	41 (24, 65)	40 (22, 64)	50 (33, 65)	41 (29, 74)	51 (30, 83)	39 (13, 59)	0.17
cT1 unspecified	49 (27, 78)	43 (25, 70)	62 (34, 123)	74 (62, 110)	30 (16, 32)	69 (34, 84)	0.047
cT2	27 (13, 47)	27 (13, 47)	34 (11, 48)	35 (9, 72)	17 (10, 112)	17 (6, 35)	0.61
cT3	28 (11, 52)	28 (12, 50)	31 (11, 62)	33 (10, 56)	32 (7, 73)	13 (7, 49)	0.82
Upstaged to pT3 or pT4, *n* (%)							
cT1 (Unspecified)	45 (15.5)	34 (15.7)	5 (11.6)	4 (36.4)	2 (18.2)	(0.0)	0.21
cT1a	62 (4.3)	51 (4.8)	6 (2.6)	4 (5.3)	1 (2.0)	(0.0)	0.35
cT1b	124 (14.5)	89 (13.9)	23 (17.7)	6 (14.0)	4 (12.1)	2 (22.2)	0.76
cT2	203 (36.9)	157 (38.1)	30 (33.7)	9 (37.5)	3 (17.6)	4 (50.0)	0.41

Among the 2573 patients with a clinical diagnosis date prior to surgery, Hispanic and AI patients with cT1 tumors had a longer time to surgery than NHW patients (median times of 56, 55, and 45 days, respectively). There was no difference in time to surgery for kidney (C649) and renal pelvis (C659) cancer. Rate of upstaging to pT3/pT4 was 4.3% in cT1a, 14.5% in cT1b, and 36.9% in cT2, and no difference was observed across racial and ethnic groups. Detailed characteristics of these patients are available in Table S2.

### 3.2 Disparities in time to surgery

**FIGURE 2:**
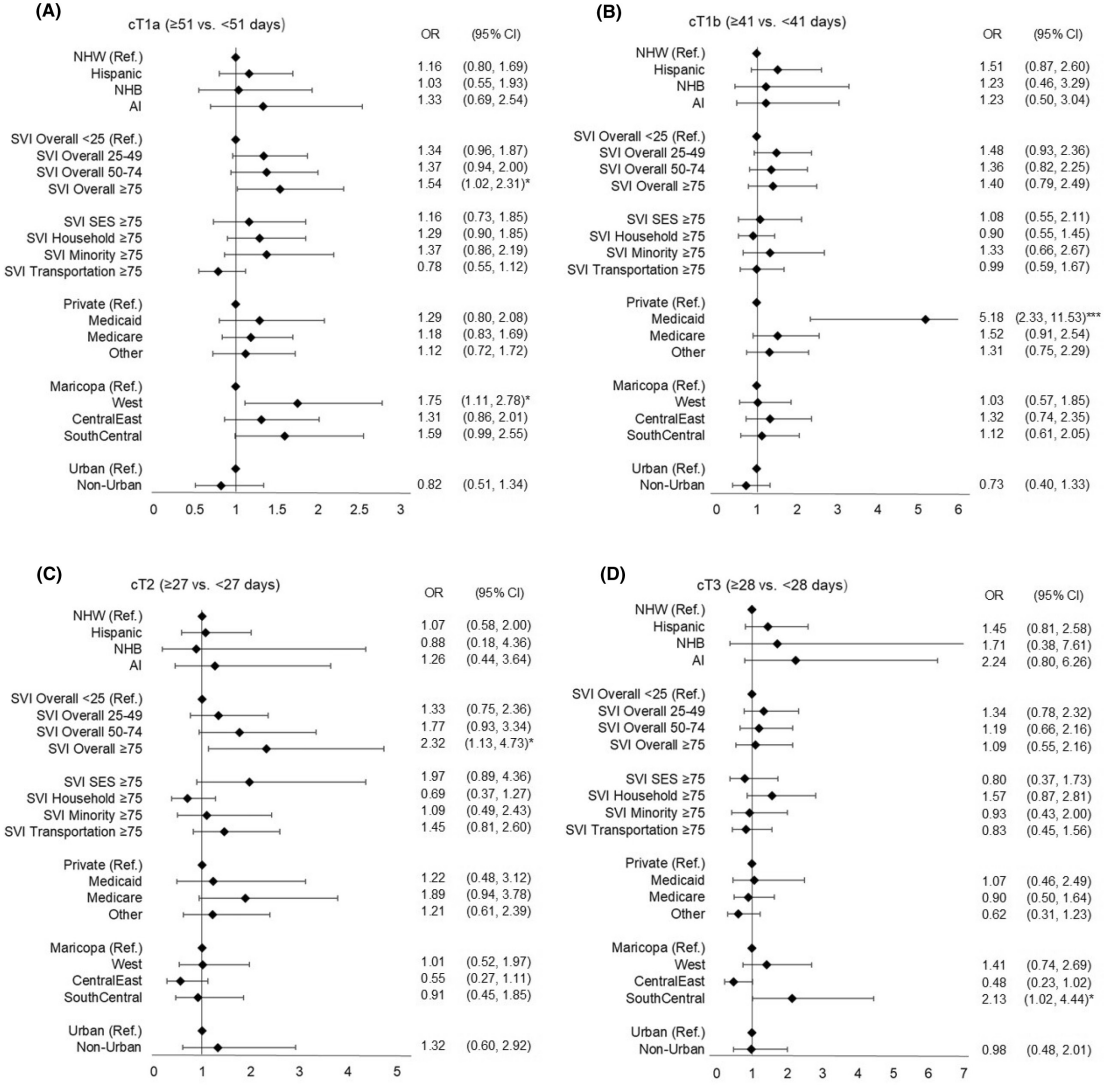
Factors associated with the longer time to surgical treatment in adjusted models. (A) cT1a (≥50 vs. <51 days), (B) cT1b (≥41 vs. <41 days), (C) cT2 (≥27 vs. <27 days), and (D) cT3 (≥28 vs. <28 days).

Hispanic ethnicity was significantly associated with a longer time to surgery for cT1a and cT1b in unadjusted models (*p* < 0.05) (Table S3). The overall SVI indicated an increased odds for a longer time to surgery among patients with cT1a, cT1b, and cT2 in the unadjusted model. In the adjusted models, the associations between Hispanic ethnicity and time to surgery were attenuated and were no longer statistically significant (Figure 2). Using different cutoff days to define longer time to surgery (>1 and >3 months to surgery after clinical diagnosis) did not change associations (Table S4). When patients were stratified by SVI categories, compared to NHW patients, Hispanic patients from areas with high concentrations of minority populations had increased odds of longer time to surgery in cT3 (OR 3.30, 95% CI: 1.11–9.84), but the association was not significant among patients from low concentrations of minority populations (*p*_Interaction_ = 0.04, Table S5). Although the interaction was not statistically significant, Hispanic patients from high SVI based on neighborhood SES in cT1b and AI patients from high SVI for housing and transportation in cT2 also had significantly increased odds of longer time to surgery (OR 2.25, 95% CI: 1.12–4.53 and OR 13.80: 1.37–139.46).

Living in high overall SVI (≥75) neighborhoods was associated with increased odds of a longer time to surgical treatment for cT1a (OR 1.54, 95% CI: 1.02–2.31) and cT2 (OR 2.32, 95% CI: 1.13–4.73). Associations between overall SVI and time to surgery were the strongest for cT2 when we assessed the association of overall SVI with >1 and >3 months to surgery. Individual SVI themes were not associated with longer time to surgery when the median time to surgery was used. However, living in neighborhoods with high SVI for housing and transportation increased odds of having >3 months to surgery after clinical diagnosis (OR 4.35, 95% CI: 1.51–12.53).

Geographical location and insurance type were also associated with time to surgery. Living in the West Arizona counties was significantly associated with a longer time to surgery for cT1a. Living in the South/Central Arizona counties increased odds of having a longer time to surgery for cT1a and cT3. Having Medicaid increased odds of a longer time to surgery for cT1b, and having Medicare increased odds of a longer time to surgery for cT1b and cT2.

### 3.3 Longer time to surgery and adverse pathology

A longer time to surgery increased odds of upstaging to any stage (OR 1.62, 95% CI: 1.02–2.57) and to pT3/pT4 (OR 1.95, 95% CI: 0.99–3.84) among patients with cT1a tumors (Table S6). Having a longer surgical time reduced odds of upstaging compared to a shorter surgical time for cT2. Living in neighborhoods with a high concentration of minority individuals increased odds of upstaging from cT1a to pT3/pT4 (OR 2.88, 95% CI: 0.99–8.37). Living in West and South/Central Arizona counties was also associated with increased odds of upstaging to pT3/pT4 in patients with cT1a tumors. A significant association between time to surgery and upstaging was not observed for patients with cT1b tumors.

### 3.4 Longer time to surgery, SVI, and survival

**FIGURE 3:**
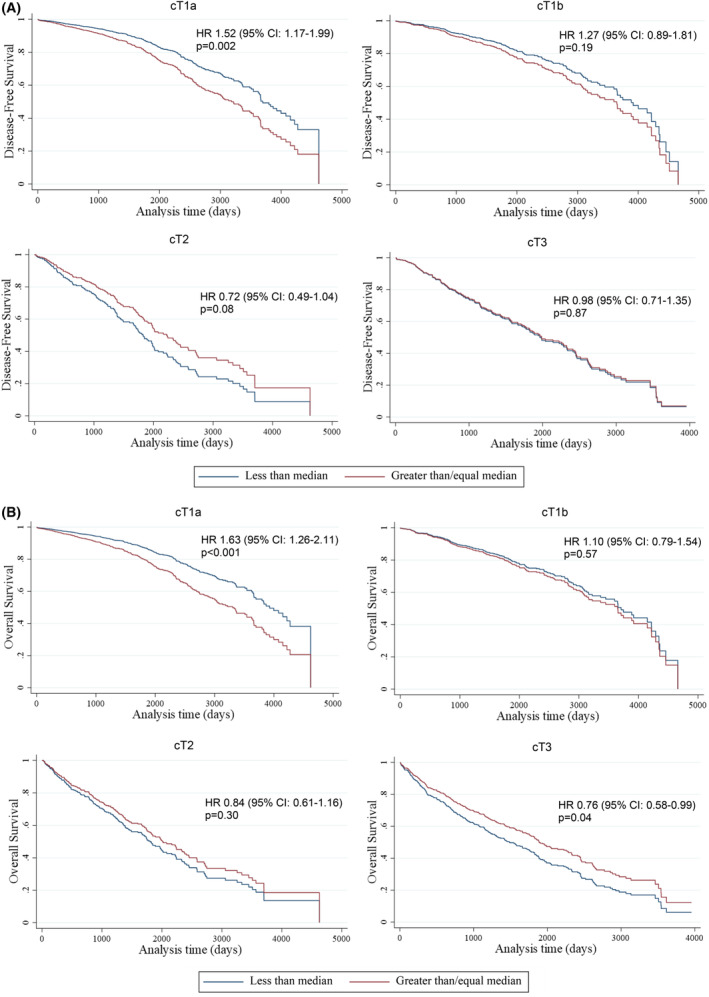
Longer time to surgery is associated with disease‐free survival (A) and overall survival (B). The Cox regression models exclude patients without a clinical diagnosis date before the surgery.

**FIGURE 4:**
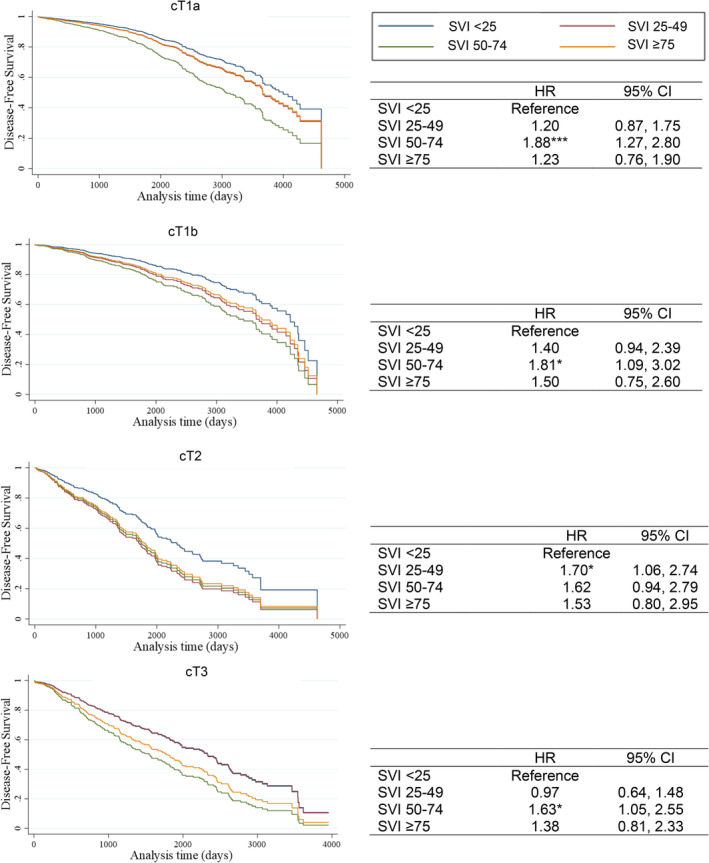
Association between overall social vulnerability index (SVI) and disease‐free survival. The Cox regression models exclude patients without a clinical diagnosis date before the surgery.

**FIGURE 5:**
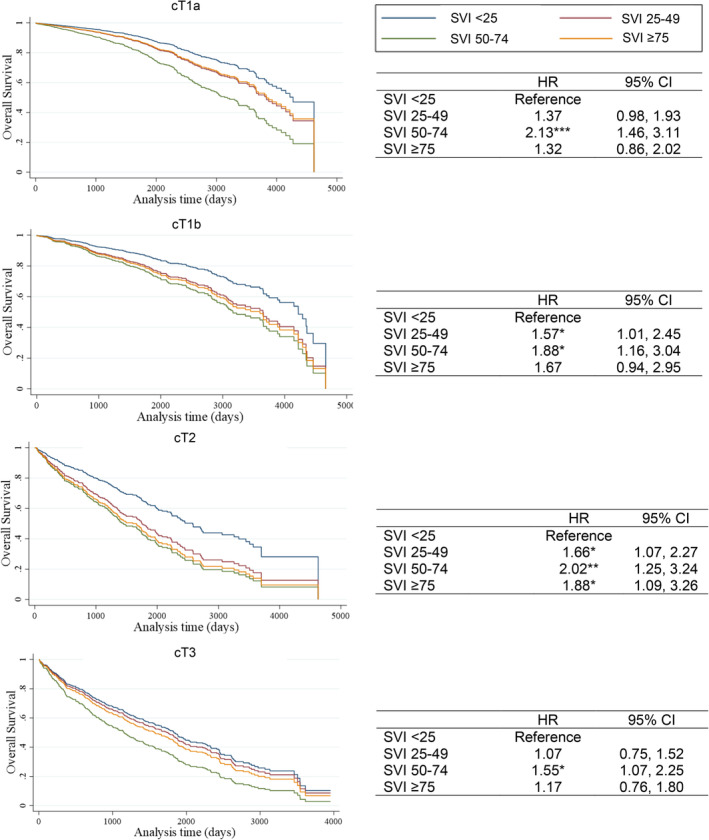
Association between overall social vulnerability index (SVI) and overall survival. The Cox regression models exclude patients without a clinical diagnosis date before the surgery.

Median time between date of surgery and date of last contact, death, or recurrence (and IQR) was 1443 days (594–2223) for cT1a, 1323 days (454–2165) for cT1b, 1164 days (554–2037) for cT2, and 1372 days (445–2293) for cT3. In the adjusted model, having a longer time to surgery was associated with worse DFS (HR 1.52, 95% CI: 1.17–1.99) and OS (HR1.63, 95% CI: 1.26–2.11) for cT1a and better OS (HR 0.76, 95% CI: 0.58–0.99) for cT3 (Figure 3). Patients living in high SVI‐score neighborhoods had poor DFS (Figure 4 **)** and OS (Figure 5 **)** across all clinical stages. Compared to patients living in neighborhoods with overall SVI <25, patients with cT2 tumors living in neighborhoods with SVI ≥75 had about 90% poorer OS (HR 1.88, 95% CI: 1.09–3.26). Patients living in neighborhoods with overall SVI 50–74 had worse DFS for cT1a (HR 1.88, 95% CI: 1.27–2.80), cT1b (HR 1.81, 95% CI: 1.09–3.02), and cT3 (HR 1.63, CI: 1.05–2.55) as well as worse OS for cT1a (HR 2.13, 95% CI: 1.46–3.11), cT1b (HR 1.88, 95% CI: 1.16–3.04), cT2 (HR 2.02, 95% CI: 1.25–3.24), and cT3 (HR 1.55, 95% CI: 1.07–2.25). Living in neighborhoods with high concentrations of racial and ethnic minorities was associated with worse DFS in cT3 (HR 2.45, 95% CI: 1.01–4.77). Individual SVI themes were not associated with OS. In these analyses, race and ethnicity were not associated with DFS or OS.

### 3.5 Assessing bias in the data on patients without a diagnosis date prior to surgery

Given the large number of cases for which a clinical diagnosis date prior to surgery was not available, we conducted an ad hoc analysis to assess whether there was potential bias in the data on outcomes in the primary analysis. A clinical diagnosis date prior to surgery was not available for 47.2%, 37.8%, and 31.3% of cases respectively for cT1, cT2, and cT3 (Table S7). Among patients with cT1 tumors, the proportion of patients without a prior diagnosis date was higher for cT1a (48.5%) than cT1b (43.8%) patients (Table S8). cT1 patients with Medicare coverage tended to have a clinical diagnosis date before surgery date. There was also geographic variation in whether this information was available. In a logistic regression analysis, cT1b patients living in West (OR 1.66, 95% CI: 1.06–2.60) and South/Central (OR 2.44, 95% CI: 1.44–4.11) counties and neighborhoods with high SVI SES scores (OR 1.79, 95% CI: 1.07–3.00) had an increased likelihood of having a clinical diagnosis date prior to surgery (Table S9). Living in neighborhoods with a high proportion of minority residents was associated with reduced odds of having a clinical diagnosis date prior to surgery (OR 0.43, 95% CI: 0.26–0.71). NHB patients with cT2 tumors and patients with cT3 tumors from Central/East Arizona counties were less likely to have a diagnosis date prior to surgery.

We further assessed whether potential bias affected results for adverse pathology and survival using patients without a clinical diagnosis date prior to surgery as a reference group and comparing them to patients with a shorter and longer time to surgery (Table S10). Among patients with cT1a tumors, the association between a longer time to surgery and upstaging became stronger (OR 2.87, 95% CI: 1.57–5.26). Having a longer time to surgery reduced odds of upstaging compared to a shorter time to surgery for cT2, but a shorter time to surgery increased odds of upstaging to pT3/pT4 compared to patients without clinical diagnosis date prior to surgery. Similarly, compared to patients without a clinical diagnosis date prior to surgery, having a shorter time to surgery was associated with worse DFS in cT2 (Table S11) and OS in cT2 and cT3 (Table S12).

### 3.6 Other factors associated with OS and DFS

In the same models including patients without clinical diagnosis date prior to surgery, we identified several other factors associated with OS and DFS. NHB patients had significantly poorer OS in cT1a (HR1.64, 95% CI: 1.05–2.54), and AI patients had significantly poorer OS in cT2 (HR 1.91, 95% CI: 1.04–3.50) than NHW patients. Patients with Medicaid coverage had significantly worse DFS for cT1a, cT2, and cT3 and OS for cT1a, cT1b, and cT3 than patients with private insurance. Patients with Medicare coverage also had significantly poorer DFS and OS for cT1a. Lastly, patients living in nonurban areas had significantly worse DFS for cT1a, cT1b, and cT3 and OS for cT1a and cT2 than patients living in urban areas.

## 4 DISCUSSION

These findings demonstrate that high neighborhood social vulnerability increases time to surgery, risk of disease progression, and mortality, which may explain some of the racial and ethnic disparity patterns in KCa. Contrary to previous studies showing that the surgery wait time of up to 3 months for early‐stage KCa may not impact pathology and survival after surgical treatment,^12^, ^14^, ^36^ our study found a longer time to surgery (>51 days) leads to adverse pathology and poor survival among patients with cT1a tumors. Our study is consistent with others showing a longer time to treatment initiation is generally associated with worse survival for KCa and other cancer types.^13^, ^15^, ^19^, ^37^ However, lack of information on potential confounding factors and bias in registry data may have affected the results of our analyses.

Racial and ethnic minority patients regularly have a longer time to cancer treatment initiation.^16^, ^19^, ^38^ During the COVID‐19 pandemic, cancer survivors from racial and ethnic minority backgrounds often delayed treatment.^39^, ^40^ However, more research is needed to understand the causal pathways linking race and ethnicity to greater time to treatment. In our study, SVI, health insurance type, and geographic region were significantly associated with time to surgery, adverse pathology, and/or survival. When these factors were included in the models, the associations between Hispanic ethnicity and time to surgery became no longer significant. This suggests that neighborhood‐level factors, geographic location, and insurance type explain the racial and ethnic disparities in time to surgical treatment. Addressing social determinants or drivers of health at the neighborhood level could improve timeliness of treatment and survival after treatment not only in racial and ethnic minority groups, but for all patients from any racial and ethnic backgrounds. However, other factors that were not included in our study, such as comorbidities, transportation, clinical complexity, individual‐level SES, cultural beliefs, institutional mistrust, health literacy, lack of financial and social support, and healthcare systems including poor care coordination and communication, may also delay the treatment of all patients.^18^, ^27^, ^41^, ^42^

A primary challenge for considering KCa disparities posed by time to surgery is the limited diversity of patients in large national databases. Racial and ethnic minority patients and some geographic areas in the United States are underrepresented in a national database commonly used for clinical quality assessment.^43^, ^44^ This complicates the generalizability of results from previous studies that assessed relationships between time to surgery and adverse pathology, as well as time to surgery and KCa survival.^12^, ^36^ A longer time to surgery for early‐stage KCa may pose differential risk across geographic locations and racial and ethnicity groups with high KCa mortality rates. Population‐based cancer registries have an advantage over hospital‐based data with better representation of racial and ethnic minority patients. For this reason, we leveraged an available population‐based cancer registry data from Arizona, a state where marked KCa disparities exist.^34^

Despite common use of population‐based and hospital‐based registry data for clinical research, both kinds of data have several issues that potentially impact the analysis results. First, many registry patients included in our study and in a previous study^36^ did not have an available clinical diagnosis date prior to their date of surgery. Renal masses are often found incidentally with imaging modalities, such as ultrasound, computed tomography (CT), and magnetic resonance imaging (MRI).^45^, ^46^ After the initial finding, patients may undergo more than one imaging assessment preoperatively to characterize the tumor (size and location of the tumor) and to plan for surgical treatment. A kidney biopsy is not often performed relying on CT and MRI for clinical diagnosis.^47^ Contrast‐enhanced CT and MRI have diagnostic capacity with high accuracies.^48^, ^49^ CT is more widely available and costs less than MRI, but MRI has capabilities of detailed characterization of renal cell carcinoma.^50^, ^51^ The pattern for the patients who had 0 days for time between clinical diagnosis and surgery may not be occurring at random and may have affected our study results showing significant associations of a shorter time to surgery with adverse pathology and survival. However, the underlying causes driving this are not clear. Second, patients, particularly those of racial and ethnic minority backgrounds, patients residing in high SVI neighborhoods, and patients with public insurance, may have had an initial abnormal finding well before their clinical diagnosis date based on imaging assessments that are reported to cancer registries, and this dataset may not capture the true extent of treatment delay. Other potential issues include lead time bias, such as patients from low SVI neighborhoods having earlier detection of tumor through imaging assessment than patients from high SVI neighborhoods, effectiveness of treatment impacting survival outcomes, and lack of detailed clinical information which may have impacted treatment and survival. Finally, some Hispanic immigrants may have gone back to their country of origin to receive care, and their treatment and outcome information may not be reported to cancer registries, resulting in underestimations of mortality rates and other estimates, a phenomenon referred to as the salmon bias.^52^, ^53^

This study identified specific geographic areas in Arizona that may benefit from targeted intervention efforts aimed at improving access to KCa care. In Arizona, many urologists specializing in KCa treatment practice in the two counties with metropolitan areas (Maricopa and Pima). Southern counties located along the US/Mexico borders have high concentrations of Hispanic populations in rural and urban areas within these counties. While Hispanic ethnicity was not associated with time to surgery, upstaging, OS, or DFS after adjusting for SVI and region/county, living in South/Central counties was associated with these outcomes. Northeastern counties in Arizona have large rural areas with AI tribal reservations. Living in nonurban areas was associated with poor OS and DFS. Racial and ethnic minority patients from rural counties may have additional challenges, but NHW also experience challenges to receive timely care. To address these disparities, interventions may be necessary to improve healthcare access in these areas with high SVI.

There are some limitations to this study. First, the sample size was small after removing cases without a clinical diagnosis date prior to surgery. Sample size, especially for racial and ethnic minority patients, was also small after stratifying by SVI. The limited sample size may have produced spurious associations and limited our ability to detect important associations. Second, despite statistical significance, effect size (OR and HR) for some association was small (<1.5) or not very large (<2.0). Developing effective public health strategies to address social drivers to health in particular geographic area or neighborhoods would be challenging without additional data. Third, most patients had to have insurance coverage before undergoing imaging assessment, and it is unknown how long the uninsured individuals had to wait to obtain insurance coverage and schedule an appointment for imaging assessment after they first experienced symptoms. Also, there may be uncontrolled confounding factors for which data were not available in the ACR yielding unexpected results. Moreover, the patients who had a small renal mass (cT1a) may have chosen to undergo active surveillance, but the data were not available for this study to confirm this clinical decision. It is possible that racial and ethnic minority patients and patients from high SVI neighborhoods are more likely to choose active surveillance, leading to greater time to surgery. Finally, some of the findings may be unique to Arizona, and may not be generalizable. The examination of the effect of neighborhood social vulnerability on disparities in time to surgical treatment in other states is warranted.

As incidence rates of KCa continue to rise in racial and ethnic minority groups, identifying factors contributing to persistent disparities in treatment and survival should remain at the forefront of research. This study showed that neighborhood‐level social vulnerability partly accounts for KCa disparities in Arizona. Patients residing in neighborhoods with high SVI had negative outcomes across all KCa clinical stages. While future research is needed to understand why patients from neighborhoods with high SVI experience worse KCa outcomes, researchers should consider designing interventions that reach these communities.

## AUTHOR CONTRIBUTIONS

**Celina I. Valencia:** Conceptualization (equal); funding acquisition (supporting); writing – original draft (equal); writing – review and editing (equal). **Patrick Wightman:** Data curation (lead); formal analysis (lead); funding acquisition (supporting); writing – original draft (supporting); writing – review and editing (supporting). **Kristin E. Morrill:** Investigation (supporting); writing – original draft (supporting); writing – review and editing (supporting). **Chiu‐Hsieh Hsu:** Formal analysis (supporting); funding acquisition (supporting); methodology (supporting). **Hina Arif‐Tiwari:** Funding acquisition (supporting); investigation (supporting). **Eric Kauffman:** Investigation (supporting); methodology (supporting). **Francine C. Gachupin:** Investigation (supporting). **Juan Chipollini:** Funding acquisition (supporting); investigation (supporting); writing – review and editing (supporting). **Benjamin R. Lee:** Writing – review and editing (supporting). **David O. Garcia:** Funding acquisition (equal); project administration (equal). **Ken Batai:** Conceptualization (equal); formal analysis (supporting); funding acquisition (equal); methodology (supporting); project administration (equal); writing – original draft (equal); writing – review and editing (equal).

## FUNDING INFORMATION

This project was supported by funding from National Cancer Institute (1R21CA248361–01 and U54CA143924) and American Cancer Society grant for a Cancer Health Equity Research Center at Minority Serving Institutions (CHERC‐MSI‐21‐167‐01‐CHERC‐MSI).

## CONFLICT OF INTEREST STATEMENT

Dr Garcia is a National Board member for the American Cancer Society Cancer Action Network (CAN). The work here does not represent the views of the ACS CAN, and is not directly related to this manuscript.

## ETHICS STATEMENT

Approval to conduct this study was obtained from the Arizona Department of Health Services Human Subject Review Board.

## Supporting information


Data S1.

## Data Availability

The KCa case data is available from the ACR after approval from the Arizona Department of Health Services.
