# Supplementary material for: Neighborhood social vulnerability and disparities in time to kidney cancer surgical treatment and survival in Arizona
Source: Cancer Med. 2024 Feb 24;13(3):e7007. doi: 10.1002/cam4.7007 (PMC10891465; doi:10.1002/cam4.7007)

**Supplementary Tables and Figure**

**Supplementary Table 1** Kidney cancer patients’ residence characterized with Social Vulnerability Index stratified by race and ethnicity.

|  | **Total** | **NHW** | **Hispanic** | **AI** | **NHB** | **Other** | **P** |
| --- | --- | --- | --- | --- | --- | --- | --- |
| Overall, n (%) |  |  |  |  |  |  |  |
| ≥75 | 896 (19.5) | 381 (11.3) | 325 (42.5) | 144 (65.5) | 34 (19.9) | 12 (16.4) | <0.001 |
| 50 - 74 | 1,035 (22.5) | 721 (21.4) | 225 (29.5) | 26 (11.8) | 50 (29.2) | 13 (17.8) |  |
| 25 - 49 | 1,258 (27.4) | 1,026 (30.5) | 137 (17.9) | 24 (10.9) | 50 (29.2) | 21 (28.8) |  |
| <25 | 1,392 (30.3) | 1,228 (36.5) | 74 (9.7) | 26 (11.8) | 37 (21.6) | 27 (37.0) |  |
| Missing | 11 (0.2) | 8 (0.2) | 3 (0.4) |  |  |  |  |
| Socioeconomic Status, n (%) |  |  |  |  |  |  |  |
| ≥75 | 852 (18.6) | 348 (10.3) | 321 (42.0) | 136 (61.8) | 35 (20.5) | 12 (16.4) | <0.001 |
| <75 | 3,740 (81.4) | 3,016 (89.7) | 443 (58.0) | 84 (38.2) | 136 (79.5) | 61 (83.6) |  |
| Household Characteristics |  |  |  |  |  |  |  |
| ≥75 | 989 (21.5) | 524 (15.6) | 288 (37.7) | 128 (58.2) | 39 (22.8) | 10 (13.7) | <0.001 |
| <75 | 3,603 (78.5) | 2,840 (84.4) | 476 (62.3) | 92 (41.8) | 132 (77.2) | 63 (86.3) |  |
| Racial and Ethnic Minority Status |  |  |  |  |  |  |  |
| ≥75 | 828 (18.0) | 328 (9.8) | 362 (47.4) | 79 (35.9) | 43 (25.1) | 16 (21.9) | <0.001 |
| <75 | 3,764 (82.0) | 3,036 (90.2) | 402 (52.6) | 141 (64.1) | 128 (74.9) | 57 (78.1) |  |
| Housing Type and Transportation |  |  |  |  |  |  |  |
| SVI Transportation ≥75 | 983 (21.4) | 556 (16.5) | 265 (34.7) | 110 (50.0) | 39 (22.8) | 13 (17.8) | <0.001 |
| SVI Transportation <75 | 3,609 (78.6) | 2,808 (83.5) | 499 (65.3) | 110 (50.0) | 132 (77.2) | 60 (82.2) |  |

**Supplementary Table 2** Characteristics of patients who had diagnosis date prior to surgery and by clinical tumor stage (n=2,573).

|  | | **cT1** | | **cT1a** | | **cT1b** | | **cT2** | | **cT3** |
| --- | --- | --- | --- | --- | --- | --- | --- | --- | --- | --- |
| **n** | | 1,797 | | 1,043 | | 566 | | 373 | | 403 |
| **Sex, n (%)** | |  | |  | |  | |  | |  |
| Male | | 1,128 (62.8) | | 639 (61.3) | | 365 (64.5) | | 283 (75.9) | | 280 (69.5) |
| Female | | 669 (37.2) | | 404 (38.7) | | 201 (35.5) | | 90 (24.1) | | 123 (30.5) |
| **Age n (%)** | |  | |  | |  | |  | |  |
| Age<50 | | 265 (14.7) | | 154 (14.8) | | 87 (15.4) | | 62 (16.6) | | 47 (11.7) |
| Age 50-59 | | 592 (32.9) | | 312 (29.9) | | 186 (32.9) | | 100 (26.8) | | 144 (35.7) |
| Age 60-69 | | 400 (22.3) | | 243 (23.3) | | 120 (21.2) | | 108 (29.0) | | 85 (21.1) |
| Age ≥70 | | 540 (30.1) | | 334 (32.0) | | 173 (30.6) | | 103 (27.6) | | 127 (31.5) |
| **Race and Ethnicity, n (%)** | |  | |  | |  | |  | |  |
| NHW | | 1,325 (73.7) | | 755 (72.4) | | 433 (76.5) | | 279 (74.8) | | 289 (71.7) |
| Hispanic | | 284 (15.8) | | 174 (16.7) | | 81 (14.3) | | 62 (16.6) | | 75 (18.6) |
| AI | | 84 (4.7) | | 48 (4.6) | | 26 (4.6) | | 18 (4.8) | | 26 (6.5) |
| NHB | | 71 (4.0) | | 45 (4.3) | | 19 (3.4) | | 7 (1.9) | | 8 (2.0) |
| Others | | 33 (1.8) | | 21 (2.0) | | 7 (1.2) | | 7 (1.9) | | 5 (1.2) |
| **Insurance Type, n (%)** | |  | |  | |  | |  | |  |
| Medicaid | | 163 (9.1) | | 101 (9.7) | | 46 (8.1) | | 23 (6.2) | | 35 (8.7) |
| Medicare | | 792 (44.1) | | 467 (44.8) | | 241 (42.6) | | 143 (38.3) | | 183 (45.4) |
| Private | | 226 (12.6) | | 362 (34.7) | | 203 (35.9) | | 50 (13.4) | | 57 (14.1) |
| Other | | 616 (34.3) | | 113 (10.8) | | 76 (13.4) | | 157 (42.1) | | 128 (31.8) |
| **Region/County, n (%)** | |  | |  | |  | |  | |  |
| Maricopa | | 1,144 (63.7) | | 694 (66.5) | | 346 (61.1) | | 221 (59.2) | | 249 (61.8) |
| West | | 229 (12.7) | | 131 (12.6) | | 81 (14.3) | | 59 (15.8) | | 64 (15.9) |
| CentralEast | | 242 (13.5) | | 132 (12.7) | | 81 (14.3) | | 52 (13.9) | | 51 (12.7) |
| SouthCentral | | 182 (10.1) | | 86 (8.2) | | 58 (10.2) | | 41 (11.0) | | 39 (9.7) |
| **Urban/non, Urban Residence, n (%)** |  | |  | |  | |  | |  |  |
| Urban | | 1,577 (87.8) | | 927 (88.9) | | 490 (86.6) | | 332 (89.0) | | 349 (86.6) |
| Non-Urban | | 220 (12.2) | | 116 (11.1) | | 76 (13.4) | | 41 (11.0) | | 54 (13.4) |
| **SVI Score, n (%)** | |  | |  | |  | |  | |  |
| SVI Overall ≥75 | | 344 (19.1) | | 208 (19.9) | | 102 (18.0) | | 70 (18.8) | | 77 (19.1) |
| SVI Overall 50-74 | | 404 (22.5) | | 216 (20.7) | | 135 (23.9) | | 87 (23.3) | | 96 (23.8) |
| SVI Overall 25-49 | | 486 (27.0) | | 293 (28.1) | | 152 (26.9) | | 114 (30.6) | | 112 (27.8) |
| SVI Overall <25 | | 556 (30.9) | | 322 (30.9) | | 175 (30.9) | | 101 (27.1) | | 117 (29.0) |
| SVI Overall Missing | | 7 (0.4) | | 4 (0.4) | | 2 (0.4) | | 1 (0.3) | | 1 (0.2) |
| SVI SES ≥75 | | 334 (18.6) | | 205 (19.7) | | 95 (16.8) | | 72 (19.3) | | 70 (17.4) |
| SVI Household ≥75 | | 408 (22.7) | | 228 (21.9) | | 135 (23.9) | | 74 (19.8) | | 90 (22.3) |
| SVI Minority ≥75 | | 293 (16.3) | | 184 (17.6) | | 76 (13.4) | | 67 (18.0) | | 70 (17.4) |
| SVI Transportation ≥75 | | 375 (20.9) | | 211 (20.2) | | 119 (21.0) | | 76 (20.4) | | 84 (20.8) |

**Supplementary Table 3** Unadjusted logistic regression analysis results for a longer time to surgery.

|  | **cT1a** | **cT1b** | **cT2** | **cT3** |
| --- | --- | --- | --- | --- |
|  | **OR (95% CI)** | **OR (95% CI)** | **OR (95% CI)** | **OR (95% CI)** |
| **Race and Ethnicity** |  |  |  |  |
| NHW | Reference | Reference | Reference | Reference |
| Hispanic | 1.48 (1.05, 2.08)* | 1.87 (1.14, 3.07)* | 1.36 (0.78, 2.39) | 1.47 (0.86, 2.50) |
| NHB | 1.16 (0.63, 2.12) | 1.55 (0.60, 3.97) | 0.73 (0.15, 3.43) | 1.87 (0.43, 8.11) |
| AI | 1.59 (0.88, 2.90) | 1.34 (0.59, 3.03) | 1.36 (0.51, 3.59) | 1.52 (0.67, 3.47) |
| Other | 0.98 (0.41, 2.35) | 0.75 (0.16, 3.45) | 0.45 (0.09, 2.42) | 1.07 (0.17, 6.69) |
| **Insurance Type** |  |  |  |  |
| Private | Reference | Reference | Reference | Reference |
| Medicaid | 1.62 (1.03, 2.54)* | 6.15 (2.82, 13.41)*** | 1.65 (0.67, 4.05) | 1.40 (0.65, 3.00) |
| Medicare | 1.28 (0.90, 1.81) | 1.55 (0.94, 2.56) | 1.97 (1.02, 3.84)* | 0.87 (0.48, 1.55) |
| Other | 1.26 (0.83, 1.94) | 1.50 (0.87, 2.57) | 1.22 (0.63, 2.35) | 0.61 (0.31, 1.19 |
| **Region/County** |  |  |  |  |
| Maricopa | Reference | Reference | Reference | Reference |
| West | 1.83 (1.25, 2.68)** | 1.11 (0.68, 1.81) | 1.38 (0.77, 2.49) | 1.48 (0.84, 2.60) |
| CentralEast | 1.42 (0.97, 2.07) | 1.36 (0.83, 2.22) | 0.78 (0.42, 1.44) | 0.57 (0.30, 1.07) |
| SouthCentral | 1.75 (1.11, 2.77)* | 1.31 (0.74, 2.30) | 1.00 (0.51, 1.97) | 2.19 (1.07, 4.48)* |
| **Urban/non-Urban Residence** |  |  |  |  |
| Urban | Reference | Reference | Reference | Reference |
| Non-Urban | 1.33 (0.90, 1.96) | 0.91 (0.56, 1.49) | 1.48 (0.76, 2.88) | 1.01 (0.56, 1.80) |
| **SVI Score** |  |  |  |  |
| SVI Overall <25 | Reference | Reference | Reference | Reference |
| SVI Overall 25-49 | 1.42 (1.03, 1.95)* | 1.57 (1.01, 2.44)* | 1.29 (0.74, 2.25) | 1.39 (0.82, 2.35) |
| SVI Overall 50-74 | 1.60 (1.12, 2.27)** | 1.67 (1.06, 2.64)* | 1.71 (0.94, 3.09) | 1.33 (0.77, 2.31) |
| SVI Overall ≥75 | 1.91 (1.33, 2.74)*** | 1.92 (1.16, 3.16)* | 2.25 (1.19, 4.27)* | 1.27 (0.70, 2.29) |
| SVI SES ≥75 | 1.23 (0.78, 1.94) | 1.21 (0.65, 2.26) | 1.93 (0.90, 4.13) | 0.85 (0.41, 1.76) |
| SVI Household ≥75 | 1.48 (1.05, 2.08)* | 1.12 (0.72, 1.74) | 0.68 (0.38, 1.21) | 1.55 (0.90, 2.66) |
| SVI Minority ≥75 | 1.35 (0.87, 2.09) | 1.64 (0.87, 3.09) | 1.13 (0.54, 2.38) | 1.16 (0.56, 2.38) |
| SVI Transportation ≥75 | 0.79 (0.55, 1.13) | 0.95 (0.58, 1.55) | 1.52 (0.86, 2.69) | 0.80 (0.45, 1.42) |

*p<0.05, **p<0.01, ***p<0.001

**Supplementary Table 4** Logistic regression analysis results for associations of race and ethnicity and SVI with having > 1 month and > 3 months to surgery after clinical diagnosis.

|  | **cT1a** | **cT1b** | **cT2** | **cT3** |
| --- | --- | --- | --- | --- |
|  | **OR (95% CI)** | **OR (95% CI)** | **OR (95% CI)** | **OR (95% CI)** |
| ***Time to Surgery > 1 month*** |  |  |  |  |
| **Race and Ethnicity** |  |  |  |  |
| NHW | Reference | Reference | Reference | Reference |
| Hispanic | 1.12 (0.72, 1.74) | 1.51 (0.84, 2.74) | 1.26 (0.67, 2.34) | 1.45 (0.81, 2.59) |
| NHB | 0.98 (0.48, 1.97) | 1.11 (0.40, 3.10) | 0.60 (0.10, 3.40) | 1.11 (0.26, 4.78) |
| AI | 1.49 (0.67, 3.32) | 1.12 (0.44, 2.83) | 1.58 (0.55, 4.57) | 2.44 (0.87, 6.82) |
| Other | 1.72 (0.56, 5.24) | 0.81 (0.17, 3.79) | 0.64 (0.12, 3.49) | 1.13 (0.18, 7.32) |
| **SVI Score** |  |  |  |  |
| SVI Overall <25 | Reference | Reference | Reference | Reference |
| SVI Overall 25-49 | 1.11 (0.77, 1.60) | 1.27 (0.79, 2.04) | 1.52 (0.85, 2.73) | 1.60 (0.92, 2.77) |
| SVI Overall 50-74 | 0.87 (0.58, 1.32) | 1.33 (0.79, 2.24) | 1.81 (0.95, 3.46) | 1.38 (0.76, 2.52) |
| SVI Overall ≥75 | 1.49 (0.92, 2.42) | 1.38 (0.75, 2.52) | 2.10 (1.02, 4.31)* | 1.47 (0.74, 2.93) |
| SVI SES ≥75 | 1.07 (0.61, 1.87) | 0.91 (0.45, 1.85) | 1.84 (0.83, 4.05) | 0.87 (0.40, 1.88) |
| SVI Household ≥75 | 1.10 (0.72, 1.68) | 0.90 (0.54, 1.50) | 0.77 (0.41, 1.43) | 1.44 (0.80, 2.57) |
| SVI Minority ≥75 | 1.50 (0.85, 2.65) | 0.98 (0.47, 2.04) | 0.93 (0.42, 2.07) | 1.08 (0.50, 2.33) |
| SVI Transportation ≥75 | 1.01 (0.66, 1.54) | 1.75 (1.00, 3.07)* | 1.32 (0.74, 2.35) | 0.88 (0.47, 1.63) |
| ***Time to Surgery > 3 months*** |  |  |  |  |
| **Race and Ethnicity** |  |  |  |  |
| NHW | Reference | Reference | Reference | Reference |
| Hispanic | 1.26 (0.80, 2.00) | 1.03 (0.49, 2.17} | 0.31 (0.06, 1.66) | 0.90 (0.24, 3.34) |
| NHB | 1.12 (0.51, 2.48) | 1.63 (0.49, 5.48) | 5.75 (0.85, 38.74) | 1.00 (1.00, 1.00) |
| AI | 0.72 (0.30, 1.72) | 1.39 (0.40, 4.82) | 3.25 (0.67, 15.77) | 4.30 (0.79, 23.38) |
| Other | 1.40 (0.45, 4.35) | 1.90 (0.21, 17.15) | NA | NA |
| **SVI Score** |  |  |  |  |
| SVI Overall <25 | Reference | Reference | Reference | Reference |
| SVI Overall 25-49 | 0.94 (0.60, 1.47) | 0.86 (0.40, 1.82) | 0.39 (0.04, 4.05) | 1.17 (0.33, 4.17) |
| SVI Overall 50-74 | 0.99 (0.60, 1.63) | 2.11 (1.04, 4.27)* | 4.60 (1.06, 19.95)* | 2.36 (0.69, 8.15) |
| SVI Overall ≥75 | 1.46 (0.87, 2.43) | 1.28 (0.56, 2.93) | 9.69 (2.15, 43.57)** | 0.62 (0.11, 3.43) |
| SVI SES ≥75 | 1.31 (0.75, 2.27) | 1.70 (0.65, 4.41) | 6.97 (1.66, 29.37)** | 0.18 (0.03, 1.20) |
| SVI Household ≥75 | 1.11 (0.72, 1.73) | 0.96 (0.48, 1.93) | 1.39 (0.44, 4.36) | 1.54 (0.47, 5.02) |
| SVI Minority ≥75 | 1.12 (0.64, 1.98) | 1.03 (0.40, 2.69) | 0.69 (0.18, 2.73) | 0.67 (0.14, 3.33) |
| SVI Transportation ≥75 | 0.98 (0.63, 1.53) | 0.86 (0.40, 1.86) | 4.35 (1.51, 12.53)** | 1.02 (0.27, 3.81) |

*p<0.05, **p<0.01, ***p<0.001, NA for categories with small sample size

**Supplementary Table 5** Logistic regression analysis results for associations of race and ethnicity with longer time to surgery (> median time) stratified by SVI (<50^th^ and ≥50^th^ percentile).

|  | **cT1a** | | **cT1b** | |
| --- | --- | --- | --- | --- |
|  | **OR (95% CI)** | **OR (95% CI)** | **OR (95% CI)** | **OR (95% CI)** |
| **Overall SVI** | ≤50 | >50 | ≤50 | >50 |
| n | 617 | 422 | 318 | 236 |
| NHW | Reference | Reference | Reference | Reference |
| Hispanic | 0.82 (0.42, 1.57) | 1.39 (0.87, 2.22) | 1.20 (0.48, 2.98) | 1.88 (0.93, 3.82) |
| NHB | 1.05 (0.40, 2.71) | 1.22 (0.52, 2.86) | 1.84 (0.43, 7.83) | 0.71 (0.17, 3.04) |
| AI | 1.27 (0.39, 4.13) | 1.35 (0.62, 2.96) | 0.77 (0.12, 5.13) | 1.55 (0.54, 4.50) |
| Other | 1.15 (0.43, 3.10) | 0.65 (0.08, 5.07) | 1.29 (0.24, 6.86) | NA |
| **SVI SES** | ≤50 | >50 | ≤50 | >50 |
| n | 597 | 442 | 318 | 245 |
| NHW | Reference | Reference | Reference | Reference |
| Hispanic | 1.04 (0.54, 2.00) | 1.27 (0.81, 2.02) | 0.88 (0.34, 2.29) | 2.25 (1.12, 4.53)* |
| NHB | 1.29 (0.48, 3.45) | 0.98 (0.43, 2.21) | 0.85 (0.21, 3.40) | 2.13 (0.48, 9.39) |
| AI | 1.41 (0.45, 4.42) | 1.36 (0.63, 2.95) | 0.76 (0.13, 4.58) | 1.68 (0.58, 4.85) |
| Other | 0.92 (0.34, 2.50) | 2.27 (0.22, 23.53) | 1.24 (0.23, 6.58) | NA |
| **SVI Household** | ≤50 | >50 | ≤50 | >50 |
| n | 547 | 492 | 290 | 273 |
| NHW | Reference | Reference | Reference | Reference |
| Hispanic | 1.05 (0.55, 2.01) | 1.36 (0.86, 2.14) | 1.13 (0.43, 2.95) | 1.81 (0.93, 3.51) |
| NHB | 0.90 (0.35, 2.35) | 1.32 (0.56, 3.11) | 1.46 (0.37, 5.82) | 1.31 (0.32, 5.35) |
| AI | 0.97 (0.30, 3.16) | 1.89 (0.87, 4.12) | 1.18 (0.16, 8.60) | 1.24 (0.45, 3.40) |
| Other | 0.84 (0.31, 2.29) | 2.28 (0.22, 23.38) | 1.18 (0.22, 6.31) | NA |
| **SVI Minority** | ≤50 | >50 | ≤50 | >50 |
| n | 397 | 642 | 220 | 344 |
| NHW | Reference | Reference | Reference | Reference |
| Hispanic | 1.35 (0.84, 2.19) | 1.09 (0.57, 2.07) | 1.50 (0.74, 3.04) | 1.37 (0.53, 3.53) |
| NHB | 1.46 (0.61, 3.50) | 0.80 (0.31, 2.04) | 2.48 (0.58, 10.65) | 0.67 (0.15, 3.05) |
| AI | 1.56 (0.69, 3.54) | 1.81 (0.57, 5.79) | 1.25 (0.42, 3.72) | 1.27 (0.24, 6.69) |
| Other | 1.15 (0.24, 5.52) | 0.98 (0.33, 2.92) | 1.19 (0.06, 22.59) | 0.83 (0.13, 5.31) |
| **SVI Transportation** | ≤50 | >50 | ≤50 | >50 |
| n | 566 | 473 | 314 | 250 |
| NHW | Reference | Reference | Reference | Reference |
| Hispanic | 0.85 (0.47, 1.53) | 1.54 (0.95, 2.50) | 1.41 (0.64, 3.11) | 1.72 (0.80, 3.68) |
| NHB | 0.88 (0.33, 2.34) | 1.47 (0.62, 3.46) | 1.77 (0.41, 7.67) | 0.70 (0.16, 2.96) |
| AI | 0.74 (0.18, 3.05) | 1.57 (0.73, 3.36) | 0.73 (0.12, 4.60) | 1.49 (0.52, 4.27) |
| Other | 1.51 (0.51, 4.47) | 0.56 (0.12, 2.69) | 1.17 (0.15, 9.05) | 0.48 (0.04, 6.48) |
|  |  |  |  |  |
|  | **cT2** | | **cT3** | |
| **Overall SVI** | ≤50 | >50 | ≤50 | >50 |
| n | 216 | 156 | 230 | 167 |
| NHW | Reference | Reference | Reference | Reference |
| Hispanic | 0.96 (0.33, 2.81) | 1.10 (0.49, 2.46) | 2.35 (0.94, 5.86) | 0.89 (0.41, 1.95) |
| NHB | 0.39 (0.03, 4.34) | 2.14 (0.18, 26.13) | 0.87 (0.14, 5.61) | NA |
| AI | 0.21 (0.02, 1.93) | 4.86 (0.87, 27.36) | 2.95 (0.19, 45.56) | 1.68 (0.54, 5.20) |
| Other | 0.47 (0.04, 5.55) | 0.27 (0.02, 3.38) | 3.50 (0.29, 42.13) | NA |
| **SVI SES** | ≤50 | >50 | ≤50 | >50 |
| n | 213 | 159 | 209 | 193 |
| NHW | Reference | Reference | Reference | Reference |
| Hispanic | 1.25 (0.43, 3.65) | 1.14 (0.52, 2.49) | 1.66 (0.64, 4.32) | 1.15 (0.55, 2.42) |
| NHB | 0.35 (0.03, 3.98) | 1.84 (0.15, 22.26) | 2.09 (0.31, 13.84) | 1.94 (0.16, 23.92) |
| AI | 0.33 (0.03, 3.28) | 2.10 (0.53, 8.33) | 3.41 (0.24, 49.26) | 1.89 (0.63, 5.70) |
| Other | 0.41 (0.04, 4.28) | 0.68 (0.05, 9.92) | 2.36 (0.14, 40.62) | 0.48 (0.04, 6.37) |
| **SVI Household** | ≤50 | >50 | ≤50 | >50 |
| n | 194 | 178 | 203 | 197 |
| NHW | Reference | Reference | Reference | Reference |
| Hispanic | 1.69 (0.52, 5.49) | 1.04 (0.49, 2.21) | 1.42 (0.55, 3.65) | 1.22 (0.57, 2.59) |
| NHB | 0.62 (0.05, 8.59) | 1.49 (0.19, 11.92) | 2.48 (0.35, 17.54) | 2.04 (0.17, 24.99) |
| AI | 0.80 (0.06, 10.66) | 1.90 (0.58, 6.25) | 6.27 (0.61, 63.94) | 1.01 (0.30, 3.35) |
| Other | 0.36 (0.04, 3.69) | 3.03 (0.16, 59.07) | NA | 2.85 (0.21, 39.22) |
| **SVI Minority** | ≤50 | >50 | ≤50 | >50 |
| n | 150 | 222 | 160 | 236 |
| NHW | Reference | Reference | Reference | Reference |
| Hispanic | 1.14 (0.51, 2.55) | 1.30 (0.43, 3.91) | 0.78 (0.37, 1.68) | 3.30 (1.11, 9.84)* |
| NHB | 1.37 (0.07, 25.04) | 0.91 (0.13, 6.33) | NA | 0.56 (0.05, 5.79) |
| AI | 1.74 (0.43, 7.03) | 0.33 (0.03, 3.32) | 1.47 (0.45, 4.74) | NA |
| Other | 0.60 (0.04, 9.02) | 0.43 (0.04, 4.43) | 0.57 (0.04, 7.24) | 1.87 (0.10, 33.92) |
| **SVI Transportation** | ≤50 | >50 | ≤50 | >50 |
| n | 208 | 162 | 223 | 179 |
| NHW | Reference | Reference | Reference | Reference |
| Hispanic | 1.43 (0.59, 3.48) | 1.25 (0.54, 2.92) | 1.66 (0.72, 3.80) | 1.17 (0.52, 2.66) |
| NHB | 0.27 (0.03, 2.68) | NA | 2.18 (0.37, 12.95) | 0.38 (0.01, 10.02) |
| AI | 0.20 (0.02, 1.75) | 13.80 (1.37, 139.46)* | 3.87 (0.29, 51.90) | 1.86 (0.59, 5.84) |
| Other | 0.94 (0.05, 16.15) | 0.32 (0.03, 3.56) | 1.03 (0.16, 6.89) | NA |

*p<0.05, **p<0.01, ***p<0.001, NA for categories with small sample size

**Supplementary Table 6** Logistic regression analysis assessing association between time to surgery and upstaging.

|  | **cT1a** | **cT1b** | **cT2** |
| --- | --- | --- | --- |
|  | **OR (95% CI)** | **OR (95% CI)** | **OR (95% CI)** |
| **n** | 721 | 484 | 351 |
| **% Upstaged to any stage** | 12% | 18% | 40% |
| ≤Median time to surgery | Reference | Reference | Reference |
| >Median time to surgery | 1.62 (1.02, 2.57)* | 0.77 (0.47, 1.27) | 0.45 (0.29, 0.72)*** |
| **% Upstaged to pT3 or pT4** | 6% | 16% | 39% |
| ≤Median time to surgery | Reference | Reference | Reference |
| >Median time to surgery | 1.95 (0.99, 3.84) | 0.82 (0.49-1.36) | 0.47 (0.29-0.74)** |
| **Race and Ethnicity** |  |  |  |
| NHW | Reference | Reference | Reference |
| Hispanic | 0.37 (0.12, 1.20) | 1.36 (0.64, 2.92) | 0.85 (0.43, 1.67) |
| NHB | 0.81 (0.10, 6.55) | 1.84 (0.48, 7.01) | 0.25 (0.03, 2.36) |
| AI | 1.15 (0.31, 4.33) | 1.23 (0.35, 4.41) | 1.80 (0.60, 5.46) |
| Other | NA | 2.71 (0.47, 15.57) | 2.94 (0.50, 17.34) |
| **Insurance Type** |  |  |  |
| Private | Reference | Reference | Reference |
| Medicaid | 0.17 (0.02, 1.39) | 0.98 (0.35, 2.78) | 2.15 (0.80, 5.75) |
| Medicare | 1.16 (0.45, 2.94) | 0.73 (0.35, 1.52) | 1.00 (0.48, 2.08) |
| Other | 0.74 (0.25, 2.24) | 0.68 (0.28, 1.64) | 0.57 (0.26, 1.25) |
| **Region/County** |  |  |  |
| Maricopa | Reference | Reference | Reference |
| West | 2.95 (1.00, 8.65)* | 1.59 (0.69, 3.67) | 0.90 (0.44, 1.85) |
| CentralEast | 1.67 (0.57, 4.85) | 1.47 (0.65, 3.30) | 0.96 (0.46, 2.01) |
| SouthCentral | 3.77 (1.43, 9.90)*** | 2.04 (0.95, 4.39) | 0.89 (0.41, 1.92) |
| **Urban/non-Urban Residence** |  |  |  |
| Urban | Reference | Reference | Reference |
| Non-Urban | 1.35 (0.48, 3.80) | 0.59 (0.24, 1.45) | 0.70 (0.30, 1.67) |
| **SVI Score** |  |  |  |
| SVI Overall <25 | Reference | Reference | Reference |
| SVI Overall 25-49 | 0.63 (0.26, 1.54) | 0.88 (0.44, 1.75) | 1.45 (0.78, 2.68) |
| SVI Overall 50-74 | 0.50 (0.17, 1.49) | 0.83 (0.39, 1.78) | 1.85 (0.94, 3.64) |
| SVI Overall ≥75 | 1.75 (0.61, 4.30) | 1.40 (0.64, 3.06) | 1.56 (0.73, 3.32) |
| SVI SES ≥75 | 1.75 (0.61, 4.97) | 1.30 (0.50, 3.39) | 0.87 (0.47, 2.50) |
| SVI Household ≥75 | 0.89 (0.36, 2.20) | 0.50 (0.24, 1.02) | 0.52 (0.80, 2.88) |
| SVI Minority ≥75 | 2.88 (0.99, 8.37) | 1.08 (0.41, 2.84) | 0.95 (0.68, 3.74) |
| SVI Transportation ≥75 | 0.73 (0.29, 1.86) | 1.49 (0.74, 3.03) | 0.79 (0.34, 1.21) |

*p<0.05, **p<0.01, ***p<0.001. Results for race and ethnicity, insurance type, region/country, urban/non-urban residence, and SVI score are from logistic regression analysis for upstaging to pT3 or pT4 not including patients without diagnosis date prior to surgery.

**Supplementary Table 7** Characteristics of patients by having diagnosis date prior to surgery (or not) and by clinical tumor stage (n=4,592).

|  | **cT1 (n=3,405)** | | | **cT2 (n=600)** | | | **cT3 (n=587)** | | |
| --- | --- | --- | --- | --- | --- | --- | --- | --- | --- |
|  | **No Prior Dx** | **Have Prior Dx** | **P** | **No Prior Dx** | **Have Prior Dx** | **P** | **No Prior Dx** | **Have Prior Dx** | **P** |
| **n (%)** | 1,608 (47.2) | 1,797 (52.8) |  | 227 (37.8) | 373 (62.2) |  | 184 (31.3) | 403 (68.7) |  |
| **Sex, n (%)** |  |  |  |  |  |  |  |  |  |
| Male | 987 (61.4) | 1,128 (62.8) | 0.40 | 156 (68.7) | 283 (75.9) | 0.06 | 134 (72.8) | 280 (69.5) | 0.41 |
| Female | 621 (38.6) | 669 (37.2) |  | 71 (31.3) | 90 (24.1) |  | 50 (27.2) | 123 (30.5) |  |
| **Age n (%)** |  |  |  |  |  |  |  |  |  |
| Age<50 | 284 (17.7) | 265 (14.7) | 0.08 | 42 (18.5) | 62 (16.6) | 0.27 | 16 (8.7) | 47 (11.7) | 0.65 |
| Age 50-59 | 483 (30.0) | 592 (32.9) |  | 52 (22.9) | 100 (26.8) |  | 72 (39.1) | 144 (35.7) |  |
| Age 60-69 | 354 (22.0) | 400 (22.3) |  | 56 (24.7) | 108 (29.0) |  | 41 (22.3) | 85 (21.1) |  |
| Age ≥70 | 487 (30.3) | 540 (30.1) |  | 77 (33.9) | 103 (27.6) |  | 55 (29.9) | 127 (31.5) |  |
| **Race and Ethnicity, n (%)** |  |  |  |  |  |  |  |  |  |
| NHW | 1,166 (72.5) | 1,325 (73.7) | 0.80 | 168 (74.0) | 279 (74.8) | 0.13 | 137 (74.5) | 289 (71.7) | 0.61 |
| Hispanic | 273 (17.0) | 284 (15.8) |  | 38 (16.7) | 62 (16.6) |  | 32 (17.4) | 75 (18.6) |  |
| AI | 78 (4.9) | 84 (4.7) |  | 7 (3.1) | 18 (4.8) |  | 7 (3.8) | 26 (6.5) |  |
| NHB | 67 (4.2) | 71 (4.0) |  | 12 (5.3) | 7 (1.9) |  | 6 (3.3) | 8 (2.0) |  |
| Others | 24 (1.5) | 33 (1.8) |  | 2 (0.9) | 7 (1.9) |  | 2 (1.1) | 5 (1.2) |  |
| **Insurance Type, n (%)** |  |  |  |  |  |  |  |  |  |
| Medicaid | 163 (10.1) | 163 (9.1) | 0.01 | 24 (10.6) | 23 (6.2) | 0.08 | 17 (9.2) | 35 (8.7) | 0.83 |
| Medicare | 641 (39.9) | 792 (44.1) |  | 71 (31.3) | 143 (38.3) |  | 84 (45.7) | 183 (45.4) |  |
| Private | 179 (11.1) | 226 (12.6) |  | 39 (17.2) | 50 (13.4) |  | 21 (11.4) | 57 (14.1) |  |
| Other | 625 (38.9) | 616 (34.3) |  | 93 (41.0) | 157 (42.1) |  | 62 (33.7) | 128 (31.8) |  |
| **Region/County, n (%)** |  |  |  |  |  |  |  |  |  |
| Maricopa | 1,145 (71.2) | 1,144 (63.7) | <0.001 | 143 (63.0) | 221 (59.2) | 0.33 | 114 (62.0) | 249 (61.8) | 0.08 |
| West | 149 (9.3) | 229 (12.7) |  | 25 (11.0) | 59 (15.8) |  | 21 (11.4) | 64 (15.9) |  |
| CentralEast | 201 (12.5) | 242 (13.5) |  | 37 (16.3) | 52 (13.9) |  | 36 (19.6) | 51 (12.7) |  |
| SouthCentral | 113 (7.0) | 182 (10.1) |  | 22 (9.7) | 41 (11.0) |  | 13 (7.1) | 39 (9.7) |  |
| **Urban/non-Urban Residence, n (%)** | | |  |  |  |  |  |  |  |
| Urban | 1,471 (91.5) | 1,577 (87.8) | <0.001 | 207 (91.2) | 332 (89.0) | 0.39 | 164 (89.1) | 349 (86.6) | 0.39 |
| Non-Urban | 137 (8.5) | 220 (12.2) |  | 20 (8.8) | 41 (11.0) |  | 20 (10.9) | 54 (13.4) |  |
| **SVI Score, n (%)** |  |  |  |  |  |  |  |  |  |
| SVI Overall ≥75 | 321 (20.0) | 344 (19.1) | 0.35 | 45 (19.8) | 70 (18.8) | 0.93 | 39 (21.2) | 77 (19.1) | 0.85 |
| SVI Overall 50-74 | 353 (22.0) | 404 (22.5) |  | 57 (25.1) | 87 (23.3) |  | 38 (20.7) | 96 (23.8) |  |
| SVI Overall 25-49 | 426 (26.5) | 486 (27.0) |  | 69 (30.4) | 114 (30.6) |  | 51 (27.7) | 112 (27.8) |  |
| SVI Overall <25 | 507 (31.5) | 556 (30.9) |  | 55 (24.2) | 101 (27.1) |  | 56 (30.4) | 117 (29.0) |  |
| SVI Overall Missing | 1 (0.1) | 7 (0.4) |  | 1 (0.4) | 1 (0.3) |  |  | 1 (0.2) |  |
| SVI SES ≥75 | 296 (18.4) | 334 (18.6) | 0.89 | 41 (18.1) | 72 (19.3) | 0.71 | 39 (21.2) | 70 (17.4) | 0.27 |
| SVI Household ≥75 | 333 (20.7) | 408 (22.7) | 0.16 | 49 (21.6) | 74 (19.8) | 0.61 | 35 (19.0) | 90 (22.3) | 0.36 |
| SVI Minority ≥75 | 319 (19.8) | 293 (16.3) | 0.007 | 38 (16.7) | 67 (18.0) | 0.70 | 41 (22.3) | 70 (17.4) | 0.16 |
| SVI Transportation ≥75 | 359 (22.3) | 375 (20.9) | 0.30 | 52 (22.9) | 76 (20.4) | 0.46 | 37 (20.1) | 84 (20.8) | 0.84 |

**Supplementary Table 8** Characteristics of patients with cT1 kidney cancer.

|  | **cT1a (n=2,027)** | | | **cT1b (n=1,007)** | | | **cT1 Unspecified (n=371)** | | |
| --- | --- | --- | --- | --- | --- | --- | --- | --- | --- |
|  | **No Prior Dx** | **Have Prior Dx** | **P** | **No Prior Dx** | **Have Prior Dx** | **P** | **No Prior Dx** | **Have Prior Dx** | **P** |
| **n (%)** | 984 (48.5) | 1,043 (51.5) |  | 441 (43.8) | 566 (56.2) |  | 183 (49.3) | 188 (50.7) |  |
| **Sex, n (%)** |  |  |  |  |  |  |  |  |  |
| Male | 605 (61.5) | 639 (61.3) | 0.92 | 275 (62.4) | 365 (64.5) | 0.49 | 107 (58.5) | 124 (66.0) | 0.14 |
| Female | 379 (38.5) | 404 (38.7) |  | 166 (37.6) | 201 (35.5) |  | 76 (41.5) | 64 (34.0) |  |
| **Age n (%)** |  |  |  |  |  |  |  |  |  |
| Age<50 | 188 (19.1) | 154 (14.8) | 0.02 | 65 (14.7) | 87 (15.4) | 0.86 | 31 (16.9) | 24 (12.8) | 0.32 |
| Age 50, 59 | 298 (30.3) | 312 (29.9) |  | 139 (31.5) | 186 (32.9) |  | 50 (27.3) | 42 (22.3) |  |
| Age 60, 69 | 229 (23.3) | 243 (23.3) |  | 91 (20.6) | 120 (21.2) |  | 34 (18.6) | 37 (19.7) |  |
| Age ≥70 | 269 (27.3) | 334 (32.0) |  | 146 (33.1) | 173 (30.6) |  | 68 (37.2) | 85 (45.2) |  |
| **Race and Ethnicity, n (%)** |  |  |  |  |  |  |  |  |  |
| NHW | 717 (72.9) | 755 (72.4) | 0.98 | 315 (71.4) | 433 (76.5) | 0.30 | 134 (73.2) | 137 (72.9) | 0.74 |
| Hispanic | 160 (16.3) | 174 (16.7) |  | 80 (18.1) | 81 (14.3) |  | 33 (18.0) | 29 (15.4) |  |
| AI | 49 (5.0) | 48 (4.6) |  | 24 (5.4) | 26 (4.6) |  | 5 (2.7) | 10 (5.3) |  |
| NHB | 41 (4.2) | 45 (4.3) |  | 19 (4.3) | 19 (3.4) |  | 7 (3.8) | 7 (3.7) |  |
| Others | 17 (1.7) | 21 (2.0) |  | 3 (0.7) | 7 (1.2) |  | 4 (2.2) | 5 (2.7) |  |
| **Insurance Type, n (%)** |  |  |  |  |  |  |  |  |  |
| Medicaid | 106 (10.8) | 101 (9.7) | 0.04 | 44 (10.0) | 46 (8.1) | 0.35 | 13 (7.1) | 16 (8.5) | 0.49 |
| Medicare | 383 (38.9) | 467 (44.8) |  | 177 (40.1) | 241 (42.6) |  | 81 (44.3) | 84 (44.7) |  |
| Private | 392 (39.8) | 362 (34.7) |  | 172 (39.0) | 203 (35.9) |  | 61 (33.3) | 51 (27.1) |  |
| Other | 103 (10.5) | 113 (10.8) |  | 48 (10.9) | 76 (13.4) |  | 28 (15.3) | 37 (19.7) |  |
| **Region/County, n (%)** |  |  |  |  |  |  |  |  |  |
| Maricopa | 713 (72.5) | 694 (66.5) | 0.01 | 316 (71.7) | 346 (61.1) | 0.001 | 116 (63.4) | 104 (55.3) | 0.22 |
| West | 85 (8.6) | 131 (12.6) |  | 46 (10.4) | 81 (14.3) |  | 18 (9.8) | 17 (9.0) |  |
| CentralEast | 119 (12.1) | 132 (12.7) |  | 56 (12.7) | 81 (14.3) |  | 26 (14.2) | 29 (15.4) |  |
| SouthCentral | 67 (6.8) | 86 (8.2) |  | 23 (5.2) | 58 (10.2) |  | 23 (12.6) | 38 (20.2) |  |
| **Urban/non, Urban Residence, n (%)** | | |  |  |  |  |  |  |  |
| Urban | 911 (92.6) | 927 (88.9) | 0.004 | 396 (89.8) | 490 (86.6) | 0.12 | 164 (89.6) | 160 (85.1) | 0.19 |
| Non, Urban | 73 (7.4) | 116 (11.1) |  | 45 (10.2) | 76 (13.4) |  | 19 (10.4) | 28 (14.9) |  |
| **SVI Score, n (%)** |  |  |  |  |  |  |  |  |  |
| SVI Overall ≥75 | 192 (19.5) | 208 (19.9) | 0.18 | 93 (21.1) | 102 (18.0) | 0.56 | 36 (19.7) | 34 (18.1) | 0.19 |
| SVI Overall 50, 74 | 208 (21.1) | 216 (20.7) |  | 100 (22.7) | 135 (23.9) |  | 45 (24.6) | 53 (28.2) |  |
| SVI Overall 25, 49 | 252 (25.6) | 293 (28.1) |  | 116 (26.3) | 152 (26.9) |  | 58 (31.7) | 41 (21.8) |  |
| SVI Overall <25 | 332 (33.7) | 322 (30.9) |  | 132 (29.9) | 175 (30.9) |  | 43 (23.5) | 59 (31.4) |  |
| SVI Overall Missing |  | 4 (0.4) |  |  | 2 (0.4) |  | 1 (0.5) | 1 (0.5) |  |
| SVI SES ≥75 | 183 (18.6) | 205 (19.7) | 0.55 | 80 (18.1) | 95 (16.8) | 0.57 | 33 (18.0) | 34 (18.1) | 0.99 |
| SVI Household ≥75 | 193 (19.6) | 228 (21.9) | 0.21 | 96 (21.8) | 135 (23.9) | 0.44 | 44 (24.0) | 45 (23.9) | 0.98 |
| SVI Minority ≥75 | 188 (19.1) | 184 (17.6) | 0.40 | 97 (22.0) | 76 (13.4) | <0.001 | 34 (18.6) | 33 (17.6) | 0.80 |
| SVI Transportation ≥75 | 211 (21.4) | 211 (20.2) | 0.50 | 110 (24.9) | 119 (21.0) | 0.14 | 38 (20.8) | 45 (23.9) | 0.46 |

**Supplementary Table 9** Factors Associated with having diagnosis date prior to surgery stratified by clinical stage.

|  | **cT1a** | **cT1b** | **cT2** | **cT3** |
| --- | --- | --- | --- | --- |
|  | **OR (95% CI)** | **OR (95% CI)** | **OR (95% CI)** | **OR (95% CI)** |
| **Race and Ethnicity** |  |  |  |  |
| NHW | Reference | Reference | Reference | Reference |
| Hispanic | 1.05 (0.81, 1.37) | 0.73 (0.50, 1.06) | 1.04 (0.63, 1.72) | 1.16 (0.69, 1.94) |
| NHB | 1.08 (0.69, 1.69) | 0.74 (0.38, 1.44) | 0.36 (0.14, 0.95)* | 0.61 (0.20, 1.87) |
| AI | 0.93 (0.59, 1.45) | 0.80 (0.43, 1.50) | 1.79 (0.69, 4.66) | 2.67 (0.99, 7.17) |
| Other | 1.26 (0.65, 2.42) | 1.92 (0.49, 7.59) | 2.34 (0.46, 11.74) | 1.03 (0.19, 5.64) |
| **Insurance Type** |  |  |  |  |
| Private | Reference | Reference | Reference | Reference |
| Medicaid | 0.96 (0.70, 1.33) | 1.01 (0.62, 1.64) | 0.63 (0.32, 1.23) | 0.81 (0.39, 1.65) |
| Medicare | 1.20 (0.94, 1.54) | 1.29 (0.90, 1.85) | 1.41 (0.84, 2.36) | 1.16 (0.71, 1.92) |
| Other | 1.12 (0.82, 1.53) | 1.36 (0.88, 2.10) | 0.74 (0.44, 1.24) | 1.36 (0.74, 2.50) |
| **Region/County** |  |  |  |  |
| Maricopa | Reference | Reference | Reference | Reference |
| West | 1.33 (0.94, 1.88) | 1.66 (1.06, 2.60)* | 1.50 (0.84, 2.68) | 1.25 (0.68, 2.29) |
| CentralEast | 1.01 (0.75, 1.37) | 1.35 (0.87, 2.09) | 0.87 (0.51, 1.47) | 0.51 (0.29, 0.88)* |
| SouthCentral | 1.27 (0.90, 1.79) | 2.44 (1.44, 4.11)*** | 1.30 (0.72, 2.33) | 1.34 (0.68, 2.65) |
| **Urban/non-Urban Residence** |  |  |  |  |
| Urban | Reference | Reference | Reference | Reference |
| Non-Urban | 1.37 (0.95, 2.00) | 1.08 (0.66, 1.75) | 1.10 (0.57, 2.13) | 1.32 (0.68, 2.54) |
| **SVI Score** |  |  |  |  |
| SVI Overall <25 | Reference | Reference | Reference | Reference |
| SVI Overall 25-49 | 1.13 (0.90, 1.43) | 0.90 (0.64, 1.28) | 0.83 (0.52, 1.33 | 1.02 (0.64, 1.65) |
| SVI Overall 50-74 | 0.98 (0.75, 1.27) | 0.91 (0.63, 1.31) | 0.88 (0.53, 1.47) | 1.06 (0.62, 1.81) |
| SVI Overall ≥75 | 1.08 (0.81, 1.44) | 0.74 (0.49, 1.12) | 0.87 (0.48, 1.54) | 0.79 (0.44, 1.41) |
| SVI SES ≥75 | 1.27 (0.91, 1.78) | 1.79 (1.07, 3.00)* | 1.17 (0.61, 2.23) | 0.77 (0.39, 1.51) |
| SVI Household ≥75 | 1.10 (0.85, 1.42) | 1.14 (0.79, 1.65) | 0.91 (0.56, 1.48) | 1.36 (0.81, 2.28) |
| SVI Minority ≥75 | 0.84 (0.61, 1.16) | 0.43 (0.26, 0.71)*** | 1.10 (0.58, 2.10) | 0.62 (0.32, 1.19) |
| SVI Transportation ≥75 | 0.86 (0.66, 1.11) | 0.75 (0.51, 1.09) | 0.85 (0.54, 1.36) | 1.11 (0.65, 1.90) |

*p<0.05, **p<0.01, ***p<0.001

**Supplementary Table 10** Logistic regression analysis results for assessing association between time to surgery and upstaging to pT3 or pT4 comparing to patients without diagnosis data prior to surgery date.

|  | **cT1a** | **cT1b** | **cT2** |
| --- | --- | --- | --- |
|  | **OR (95% CI)** | **OR (95% CI)** | **OR (95% CI)** |
| **% Upstaged to pT3 or pT4** | 6% | 16% | 39% |
| No prior diagnosis date | Reference | Reference | Reference |
| ≤Median time to surgery | 1.40 (1.70, 2.80) | 1.54 (0.96, 2.47) | 1.77 (1.15, 2.71)** |
| >Median time to surgery | 2.87 (1.57, 5.26)*** | 1.31 (0.81, 2.13) | 0.85 (0.54, 1.35) |

*p<0.05, **p<0.01, ***p<0.001. Logistic regression analysis adjusting for age, sex, race and ethnicity, insurance type, region/country, urban/non-urban residence, and SVI score are from

**Supplementary Table 11** Factors associated with disease free survival stratified by clinical tumor stage including patients without diagnosis date prior to surgery as reference.

|  | **cT1a** | **cT1b** | **cT2** | **cT3** |
| --- | --- | --- | --- | --- |
|  | **HR (95% CI)** | **HR (95% CI)** | **HR (95% CI)** | **HR (95% CI)** |
| **Surgical Wait Time** |  |  |  |  |
| No prior diagnosis date | Reference | Reference | Reference | Reference |
| ≤Median time to surgery | 0.81 (0.65, 1.02) | 0.69 (0.50, 0.94)* | 1.52 (1.07, 2.17)* | 1.04 (0.76, 1.42) |
| >Median time to surgery | 1.22 (0.97, 1.53) | 0.88 (0.66, 1.18) | 1.20 (0.84, 1.72) | 1.06 (0.78, 1.44) |
| **Race and Ethnicity** |  |  |  |  |
| NHW | Reference | Reference | Reference | Reference |
| Hispanic | 0.97 (0.72, 1.30) | 1.11 (0.75, 1.63) | 0.76 (0.49, 1.20) | 0.98 (0.66, 1.47) |
| NHB | 1.60 (0.99, 2.57) | 0.85 (0.41, 1.77) | 1.50 (0.74, 3.06) | 1.00 (0.46, 2.17) |
| AI | 1.08 (0.67, 1.75) | 1.14 (0.62, 2.11) | 1.97 (0.95, 4.09) | 1.43 (0.76, 2.71) |
| Other | 0.72 (0.27, 1.93) | 0.48 (0.07, 3.45) | 1.14 (0.35, 3.67) | 2.57 (0.78, 8.52) |
| **Insurance Type** |  |  |  |  |
| Private | Reference | Reference | Reference | Reference |
| Medicaid | 1.90 (1.30, 2.78)*** | 1.70 (0.94, 3.07) | 2.13 (1.13, 4.02)* | 1.83 (1.09, 3.09)* |
| Medicare | 1.37(1.05, 1.78)* | 1.27 (0.89, 1.80) | 1.15 (0.74, 1.78) | 1.16 (0.82, 1.26) |
| Other | 1.54 (1.08, 2.19)* | 1.29 (0.81, 2.04) | 1.46 (0.91, 2.36) | 1.37 (0.88, 1.16) |
| **Region/County** |  |  |  |  |
| Maricopa | Reference | Reference | Reference | Reference |
| West | 1.09 (0.78, 1.51) | 0.91 (0.61, 1.37) | 0.59 (0.35, 1.00)* | 0.53 (0.33, 0.85)** |
| CentralEast | 0.95 (0.69, 1.31) | 0.80 (0.53, 1.23) | 0.73 (0.43, 1.23) | 0.84 (0.56, 1.26) |
| SouthCentral | 1.50 (0.99, 2.26) | 1.26 (0.71, 2.22) | 1.68 (1.00, 2.80)* | 0.70 (0.42, 1.16) |
| **Urban/non-Urban Residence** |  |  |  |  |
| Urban | Reference | Reference | Reference | Reference |
| Non-Urban | 1.56 (1.10, 2.23)* | 1.60 (1.03, 2.50)* | 1.50 (0.86, 2.60) | 1.67 (1.07, 2.59)* |
| **SVI Score** |  |  |  |  |
| SVI Overall <25 | Reference | Reference | Reference | Reference |
| SVI Overall 25-49 | 1.32 (1.02, 1.70)* | 1.18 (0.85, 1.64) | 1.40 (0.95, 2.07) | 1.14 (0.82, 1.60) |
| SVI Overall 50-74 | 1.55 (1.17, 2.06)** | 1.31 (0.92, 1.87) | 1.25 (0.80, 1.94) | 1.73 (1.18 2.53)* |
| SVI Overall ≥75 | 1.55 (1.14, 2.11)** | 1.11 (0.72, 1.73) | 0.99 (0.58, 1.71) | 1.23 (0.80, 1.89) |
| SVI SES ≥75 | 0.88 (0.61, 1.26) | 0.87 (0.52, 1.46) | 1.02 (0.55, 1.89) | 0.78 (0.44, 1.39) |
| SVI Household ≥75 | 1.25 (0.94, 1.65) | 1.16 (0.81, 1.66) | 0.88 (0.55, 1.41) | 1.12 (0.77, 1.64) |
| SVI Minority ≥75 | 1.42 (0.99, 2.02) | 1.37 (0.83, 2.27) | 1.06 (0.58, 1.95) | 1.43 (0.84, 2.44) |
| SVI Transportation ≥75 | 1.00 (0.75, 1.33) | 0.83 (0.56, 1.23) | 1.07 (0.69, 1.64) | 0.99 (0.67, 1.46) |
| **Surgery type ^1^** |  |  |  |  |
| Surgery Type Unknown | Reference | Reference |  |  |
| Total or Radical Nephrectomy | 0.99 (0.48, 2.04) | 0.60 (0.34, 1.07) |  |  |
| Partial Nephrectomy | 0.64 (0.31, 1.33) | **0.33 (0.17, 0.62)***** | 1.63 (0.94, 2.83) | 0.62 (0.36, 1.07) |
| Local Ablation | 1.16 (0.55, 2.41) | 0.77 (0.36, 1.07) |  |  |
| **Sex** |  |  |  |  |
| Male | Reference | Reference | Reference | Reference |
| Female | 0.68 (0.55, 0.83)*** | 0.71 (0.54, 0.92)* | 0.91 (0.64, 1.30) | 0.93 (0.36, 1.07) |
| **Age** |  |  |  |  |
| Age<50 | 0.40 (0.26, 0.63)*** | 0.34 (0.18, 0.64)*** | 0.57 (0.31, 1.06) | 0.92 (0.53, 1.58) |
| Age 50-59 | 0.66 (0.48, 0.90)*** | 0.65 (0.41, 1.02) | 0.70 (0.45, 1.08) | 0.71 (0.47, 1.06) |
| Age 60-69 | Reference | Reference | Reference | Reference |
| Age≥70 | 1.54 (1.22, 1.94)*** | 1.64 (1.22, 2.20)*** | 1.79 (1.22, 2.62)** | 1.42 (1.04, 1.94) |

*p<0.05, **p<0.01, ***p<0.001. The reference group used in analysis for cT2 and cT3 includes Unknown, Total and Radical Nephrectomy, and Local Ablation.

**Supplementary Table 12** Factors associated with overall survival stratified by clinical tumor stage including patients without diagnosis date prior to surgery as reference.

|  | **cT1a** | **cT1b** | **cT2** | **cT3** |
| --- | --- | --- | --- | --- |
|  | **HR (95% CI)** | **HR (95% CI)** | **HR (95% CI)** | **HR (95% CI)** |
| **Surgical Wait Time** |  |  |  |  |
| No prior Dx date | Reference | Reference | Reference | Reference |
| <Median wait time | 0.78 (0.63, 0.98)* | 0.79 (0.59, 1.07) | 1.48 (1.07, 2.04)* | 1.36 (1.04, 1.77)* |
| >Median wait Time | 1.23 (0.99, 1.53) | 0.92 (0.69, 1.22) | 1.33 (0.96, 1.83) | 1.05 (0.80, 1.37) |
| **Race and Ethnicity** |  |  |  |  |
| NHW | Reference | Reference | Reference | Reference |
| Hispanic | 1.01 (0.76, 1.34) | 1.18 (0.82, 1.70) | 0.82 (0.55, 1.22) | 1.13 (0.83, 1.54) |
| NHB | 1.64 (1.05, 2.54)* | 1.06 (0.55, 2.06) | 1.36 (0.67, 2.73) | 0.95 (0.46, 1.95) |
| AI | 1.12 (0.70, 1.77) | 1.20 (0.66, 2.17) | 1.91 (1.04, 3.50)* | 1.26 (0.75, 2.15) |
| Other | 0.70 (0.26, 1.90) | 0.39 (0.05, 2.84) | 0.20 (0.03, 1.48) | 1.45 (0.53, 3.97) |
| **Health Insurance** |  |  |  |  |
| Private | Reference | Reference | Reference | Reference |
| Medicaid | 2.08 (1.43, 3.02)*** | 1.75 (1.01, 3.03)* | 1.62 (0.91, 2.86) | 1.61 (1.06, 2.45)* |
| Medicare | 1.51 (1.16, 1.97)** | 1.38 (0.97, 1.96) | 1.25 (0.84, 1.86) | 0.98 (0.73, 1.32) |
| Other | 1.61 (1.14, 2.29)** | 1.41 (0.91, 2.18) | 1.35 (0.87, 2.09) | 1.28 (0.89, 1.83) |
| **Region/County, n (%)** |  |  |  |  |
| Maricopa | Reference | Reference | Reference | Reference |
| West | 1.09 (0.79, 1.49) | 0.99 (0.68, 1.44) | 0.62 (0.40, 0.98)* | 0.60 (0.41, 0.88)** |
| CentralEast | 0.95 (0.69, 1.29) | 0.80 (0.53, 1.21) | 0.78 (0.50, 1.21) | 0.80 (0.56, 1.14) |
| SouthCentral | 1.42 (0.94, 2.14) | 1.41 (0.84, 2.37) | 1.82 (1.19, 2.79)** | 0.71 (0.47, 1.07) |
| **Urban/non-Urban Residence** |  |  |  |  |
| Urban | Reference | Reference | Reference | Reference |
| Non-Urban | 1.52 (1.08, 2.14)* | 1.45 (0.94, 2.22) | 1.64 (1.03, 2.61)* | 1.40 (0.96, 2.05) |
| **SVI Score** |  |  |  |  |
| SVI Overall <25 | Reference | Reference | Reference | Reference |
| SVI Overall 25-49 | 1.35 (1.06, 1.72)* | 1.19 (0.86, 1.65) | 1.41 (0.99, 2.01) | 1.16 (0.87, 1.55) |
| SVI Overall 50-74 | 1.62 (1.24, 2.13)*** | 1.35 (0.80, 1.86) | 1.45 (0.98, 2.15) | 1.63 (1.18, 2.24)** |
| SVI Overall ≥75 | 1.54 (1.14, 2.08)** | 1.22 (0.80, 1.86) | 1.14 (0.71, 1.81) | 1.05 (0.73, 1.52) |
| SVI SES ≥75 | 0.88 (0.62, 1.25) | 0.95 (0.58, 1.55) | 0.88 (0.51, 1.52) | 0.84 (0.54, 1.32) |
| SVI Household ≥75 | 1.28 (0.98, 1.68) | 1.13 (0.81, 1.58) | 1.02 (0.69, 1.51) | 1.23 (0.90, 1.67) |
| SVI Minority ≥75 | 1.28 (0.91, 1.80) | 1.32 (0.82, 2.13) | 1.41 (0.83, 2.39) | 1.10 (0.71, 1.70) |
| SVI Transportation ≥75 | 1.03 (0.78, 1.36) | 0.90 (0.62, 1.30) | 0.99 (0.68, 1.44) | 0.89 (0.64, 1.23) |
| **Surgery type** |  |  |  |  |
| Surgery Type Unknown | Reference | Reference |  |  |
| Total or Radical Nephrectomy | 0.93 (0.47, 1.85) | 0.63 (0.35, 1.12) |  |  |
| Partial Nephrectomy | 0.60 (0.30, 1.19) | 0.34 (0.18, 0.64)*** | 1.16 (0.66, 2.01) | 0.61 (0.37, 1.00) |
| Local Ablation | 1.14 (0.57, 2.29) | 0.88 (0.43, 1.82) |  |  |
| **Sex** |  |  |  |  |
| Male | Reference | Reference | Reference | Reference |
| Female | 0.71 (0.58, 0.87)*** | 0.77 (0.59, 0.99)* | 0.85 (0.62, 1.17) | 0.86 (0.68, 1.10) |
| **Age** |  |  |  |  |
| Age<50 | 0.35 (0.22, 0.54)*** | 0.42 (0.23, 0.74)** | 0.73 (0.43, 1.23) | 0.76 (0.49, 1.19) |
| Age 50-59 | 0.58 (0.43, 0.80)*** | 0.70 (0.45, 1.09) | 0.83 (0.56, 1.23) | 0.76 (0.55, 1.06) |
| Age 60-69 | Reference | Reference | Reference | Reference |
| Age≥70 | 1.48 (1.18, 1.84)*** | 1.71 (1.28, 2.27)*** | 1.66 (1.18, 2.33)** | 1.24 (0.95, 1.64) |

*p<0.05, **p<0.01, ***p<0.001. The reference group used in analysis for cT2 and cT3 includes Unknown, Total and Radical Nephrectomy, and Local Ablation.

**Supplementary Figure 1** Diagram showing sample selection.


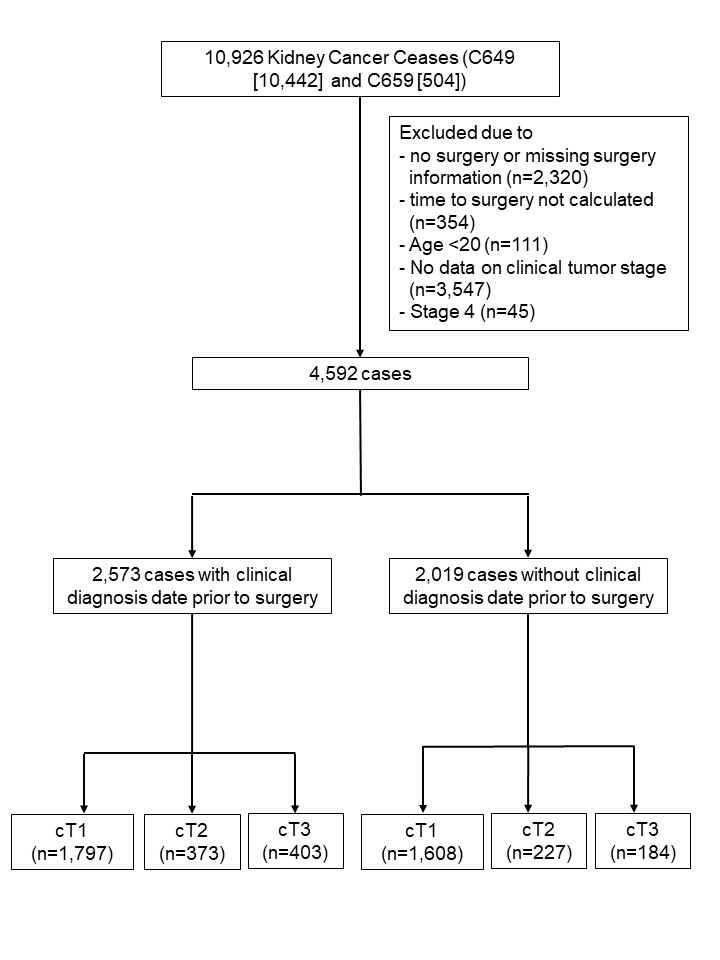

Supplement: Supplementary file 1 — Data S1. [file CAM4-13-e7007-s001.docx]
